# Concurrent assessment of neurometabolism and brain hemodynamics to characterize the functional brain response to psychotropic drugs: an S-ketamine study

**DOI:** 10.1177/0271678X251399023

**Published:** 2025-11-30

**Authors:** Daphne E Boucherie, Liesbeth Reneman, Jan Booij, Rogier Immink, Markus W Hollmann, Anouk Schrantee

**Affiliations:** 1 Department of Radiology and Nuclear Medicine, Amsterdam University Medical Center, Amsterdam, the Netherlands; 2 Department of Anesthesiology, Amsterdam University Medical Center, Amsterdam, the Netherlands

**Keywords:** S-ketamine, dissociation, pharmacological MRI, functional MRS, psychopharmacology

## Abstract

Neuroimaging techniques offer valuable insights for understanding pharmacological treatment effects in neuropsychiatric disorders. Here, we present a novel approach that simultaneously assesses hemodynamic and neurometabolic brain responses to psychotropic drugs using interleaved pharmacological magnetic resonance imaging (phMRI) and pharmacological magnetic resonance spectroscopy (phMRS). This method was tested using a double-dose, placebo-controlled, randomized, crossover design using S-ketamine as the pharmacological agent. We acquired 7 Tesla phMRI and phMRS data to evaluate time- and dose-dependent effects of S-ketamine in 32 healthy controls. S-ketamine elicited robust phMRI responses in the dorsofrontal, cingulate, and insular cortices, which correlated with glutamate and opioid receptor maps and subjective dissociation scores. These hemodynamic changes were paralleled by increases in glutamate and lactate, especially at higher doses. Furthermore, accuracy in predicting received condition (placebo, a low, or a high S-ketamine dose) increased when combining both techniques. Here, we show for the first time that concurrent phMRI and phMRS assessments provide important complementary insights into the functional brain response to pharmacological interventions.

## Introduction

The development of pharmacological treatments for neuropsychiatric disorders is hindered by the lack of reliable biomarkers to assess whether a drug effectively targets the intended neural pathways or neurotransmitter systems. Imaging techniques that can non-invasively measure the brain’s response to medication are therefore essential.^1^ Pharmacological magnetic resonance imaging (phMRI) is a promising technique in this regard, due to its non-invasive nature, wide availability, good spatial resolution and whole-brain coverage. PhMRI measures hemodynamic changes in response to pharmacological stimulation of a target neurotransmitter system. In short, after oral or intravenous administration of a drug, it binds to its specific target, thereby (de)activating the neurons on which the respective receptor or transporter is located. Changes in neuronal activity then induce a cascade of neurotransmitter and metabolite release, accompanied by the release of vasoactive substances from neurons and astrocytes.^2,3^ This subsequently induces changes in local cerebral blood flow (CBF), volume (CBV) and oxygenation, an intricate process known as “neurovascular coupling”.^4^ These hemodynamic changes can subsequently be measured with various MRI techniques, such as blood-oxygen-level-dependent (BOLD) functional MRI (fMRI). As such, phMRI can serve as a proxy for, and provide an insight into, neurotransmitter function,^5–7^ and is ideally suited for drug discovery studies, as well as treatment monitoring and prediction.

Despite its considerable promise, interpreting phMRI data is challenging due to multiple complex factors influencing the phMRI signal. As phMRI relies on hemodynamic signals, non-neuronal processes, such as direct effects of psychotropic drugs on the vasculature, can affect the phMRI signal. To more directly assess the effects of medication at the neurometabolic level, proton magnetic resonance spectroscopy (^1^H-MRS) can be employed, which provides information about metabolic processes occurring within tissues. Specifically, functional MRS (fMRS), in which dynamic scans are acquired at short time intervals of several seconds, allows for the assessment of acute changes in neurometabolite levels in response to neuronal activation.^8–10^

Here, we introduce a novel approach called pharmacological MRS (phMRS), which applies fMRS in the context of psychopharmacology to directly measure neurometabolite changes over time, in response to a psychotropic drug challenge. PhMRS can be used to detect changes in metabolites like glutamate, lactate, aspartate, and glucose. Glutamate is the primary excitatory neurotransmitter, orchestrating the majority of neural activity.^11,12^ It also governs the hemodynamic response to increased neuronal activity,^13^ and its turnover is tightly linked to brain energetics.^14^ Additionally, lactate, aspartate, and glucose are key components of the neurometabolic cycle.^15^

**Figure 1.**
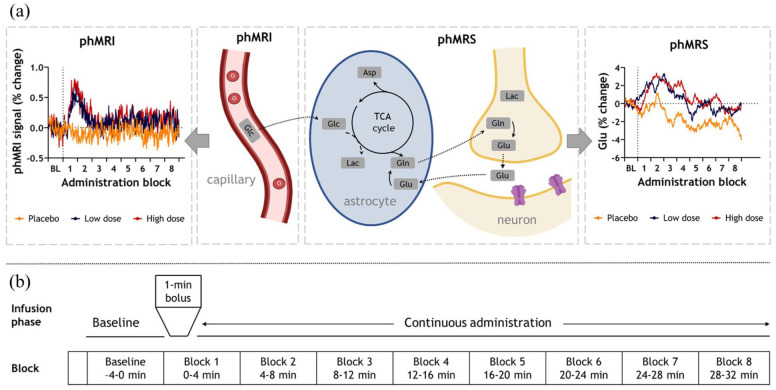
phMRI and phMRS results and acquisition scheme. (a) *Left* phMRI: percent change in BOLD signal from baseline, extracted from the anterior cingulate cortex (ACC) MRS voxel, is visualized per condition. *Middle*: Schematic overview of complementary information provided by phMRI and phMRS. *Right*: phMRS: percent change in glutamate (Glu) from baseline is visualized per condition. (b) Schematic representation of phMRI-phMRS sessions and allocation of blocks for analysis. After a 4-minute baseline acquisition (two minutes of data at the start was discarded), S-ketamine or placebo was administered and eight 4-minute infusion blocks were acquired. phMRI: pharmacological magnetic resonance imaging; BL: baseline; BOLD: blood-oxygen-level-dependent; min: minute; MRS: magnetic resonance spectroscopy.

We aimed to gain a more comprehensive understanding of the functional brain response to medication, by simultaneously assessing neurometabolic and hemodynamic contributions. Therefore, we developed and evaluated an interleaved phMRI-phMRS protocol, which concurrently measures hemodynamic and neurometabolic changes in response to drug administration (Figure 1(a)). We tested this approach using a double-dose, placebo-controlled, randomized crossover design with S-ketamine as the pharmacological agent. S-ketamine, a non-competitive N-methyl-D-aspartate (NMDA) receptor antagonist,^16^ exerts its effects through a complex cascade of neuronal interactions. By antagonizing NMDA receptors on gamma-aminobutyric acid (GABA)-ergic inhibitory interneurons,^17^ S-ketamine disrupts their inhibitory tone on excitatory pyramidal neurons. This disinhibition triggers a surge in glutamate release, which in turn activates various post-synaptic receptors, including glutamatergic AMPA receptors, as well as cholinergic and monoaminergic receptors.^18,19^ In the context of psychiatry, S-ketamine is used as treatment for treatment-resistant depression^20^ and as a pharmacological model for psychosis.^21^ Previous studies have demonstrated robust increases in BOLD signals in prefrontal, cingulate, and insular cortices and the thalamus,^22,23^ with good test-retest reliability.^24^ We assessed time- and dose-dependent effects of S-ketamine on the hemodynamic response and neurometabolite dynamics, and assessed their association with subjective effects. We also examined which receptor targets contribute to these effects by assessing correlations between phMRI responses and template receptor maps.

## Methods

The study was registered in the Dutch Trial Register (NL8994). A pre-registration for analyses was done at Open-Science Framework (https://osf.io/6hzte). All procedures were approved by the medical ethics committee (NL74447.018.20) of the Amsterdam University Medical Center prior to the start of the study and were conducted in accordance with the Declaration of Helsinki. All subjects gave written informed consent.

### Participants

We recruited healthy volunteers with the following inclusion criteria: 18–55 years of age; BMI 18.5–25 kg/m^2^. Female participants were required to use oral contraceptives, and all sessions were scheduled during active pill phases to reduce hormonal variability.^25^ Participants with a (history of) psychiatric treatment, having first-degree relatives with (a history of) schizophrenia and major depression disorder, (a history of) neurological disorders or concussion with loss of consciousness, contraindications for S-ketamine administration or 7T MRI, and (a history of) drug or alcohol dependence were excluded. Previous recreational ketamine use was not an exclusion criterion, but a minimum abstinence period of one month was required. Participants were recruited via flyers and subject recruitment websites.

### Study design

We conducted a single-blind, double-dose, placebo-controlled, randomized, crossover study. Participants visited the laboratory site four times, once for a screening visit, and three times for an MRI session. During the screening visit, participants were screened for (history of) psychiatric disorders (Mini International Neuropsychiatric Interview; M.I.N.I)), alcohol or drug dependence (Alcohol, Smoking, and Substance Involvement Screening Test; ASSIST-Lite), and underwent a physical examination during which height, weight, heart rate and blood pressure were measured. At the start of all three MRI sessions, subjects were screened using a urine test for recreational drug use and for pregnancy (for women). Participants were randomly assigned to one of six possible administration orders using a balanced randomization scheme. An intravenous line was placed by a resident anesthesiologist for S-ketamine administration and blood sampling. Participants received one of two sub-anesthetic doses of S-ketamine or placebo intravenously. The administration consisted of a bolus (1 min), followed by a continuous administration (~40 min) (Figure 1(b)) to keep plasma levels stable throughout the remainder of the imaging sessions (placebo: NaCl 0.9%; low dose: 0.11 mg/kg + 0.1 mg/kg/h; total dose ≈0.17 mg/kg; high dose: 0.22 mg/kg + 0.2 mg/kg/h; total dose ≈0.33 mg/kg). The dose was determined based on prior studies.^16^ To monitor subjective effects of S-ketamine, the Clinician-Administered Dissociative States Scale (CADSS)^26^ was administered before and immediately after the medication administration (participants were instructed to report on experienced dissociation during the administration period). Following the MRI scan, a blood sample was collected to determine S-ketamine and nor-S-ketamine blood plasma concentrations.

Participants refrained from psychotropic drugs, and recreational substances for a week before each session, from alcohol 24 hours before each session, and from caffeine on session days. Sessions were scheduled at the same time of day with at least a 7-day gap between them.

### Acquisition

Data was acquired on a 7T MR system (Philips, Best, the Netherlands) with a dual-channel transmit and 32-channel receive coil (Nova Medical Inc., Wilmington, MA, USA). A T1-weighted (T1w) structural scan was obtained (TR/TE/FA = 5 ms/2 ms/7°; FOV(APxFHxRL) = 246 × 246 × 180 mm^3^; voxel size = 0.85 × 0.85 × 1 mm^3^), followed by an interleaved fMRI/fMRS protocol (total scan duration 38 min). fMRI and fMRS were interleaved using a scan switching framework,^28^ in which scanner software alternates between independently defined scan modules by suspending and resuming their execution in real time. In our setup,^10^ this involved switching between volume TRs of the 3D-EPI fMRI sequence and signal averages (NSA) of the single-voxel sLASER fMRS acquisition within a combined dynamic scan time of 5.1 s. To account for the different shim requirements of fMRI and fMRS, we used a static higher-order shim set complemented by dynamically updated linear shim terms that alternated in synchrony with the scan switching, ensuring optimal field homogeneity for each modality.^27^ 3D-EPI fMRI: TR/TE/FA = 31/20 ms/10°, shot interval = 1600 ms, voxel size = 1.875 × 1.875 × 2.09 mm^3^, FOV = 240 × 240 × 136.5 mm^3^, 446 volumes; a top-up scan with opposing phase encoding (4 volumes) was obtained for distortion correction. Single-voxel sLASER fMRS: TR/TE = 3500/36 ms; bandwidth = 3 kHz; number of points = 1024; ACC volume of interest (VOI) size = 30 × 20 × 15 mm^3^; 448 transients; VAPOR water-suppression. The phMRS voxel was positioned in the dorsal anterior cingulate cortex (ACC), with the anterior side of the voxel in line with the genu of the corpus callosum and voxel orientation aligned with the corpus callosum. The voxel placement and overlap for all participants is visualized in Figure 3(a) (inset). The selection of the dorsal ACC was motivated by prior static MRS studies suggesting ketamine’s effect on glutamate resonances in this brain region.^29–31^ Participants were instructed to focus on a fixation cross and let their minds wander. Heart rate was measured continuously during the scan session using a peripheral pulse unit (PPU).

### Processing and analysis

#### PhMRI

PhMRI data were preprocessed using in-house scripts and fMRIprep v20.1.1,^32^ which is based on Nipype 1.4.2^33^ (see Supplemental Materials). Volumes with framewise displacement (FD)>1 mm were identified and added to the first-level analyses as confound regressors to perform motion scrubbing. First-level GLM analyses divided the data into nine blocks of 48 volumes (~4 min) in a pseudo-block design (similar to Deakin et al.^22^; McMillan et al.^23^). The first block modeled the baseline (no medication), followed by eight blocks modeling continuous administration. Each administration block was contrasted with baseline, generating eight first-level contrasts per subject per session using FSL-FEAT^34,35^ (Supplemental Figure S1). Prior research has shown that gamma-variate models (or ‘signal models’^23,24^) are more sensitive to capture the hemodynamic response to S-ketamine compared to pseudo-block designs. Despite this, we opted for the pseudo-block design to characterize changes at numerous independent time points and to maintain consistency across both phMRI and phMRS analyses. This is particularly important because, while we have knowledge of the hemodynamic response function, the response function of neurometabolism is less established.^9,10,36^ Nonetheless, we additionally analyzed our data using a signal model, results from which are provided in the Supplemental Materials.

#### PhMRS

PhMRS data processing and dynamic fitting was done in FSL-MRS^37^ (version 2.1.13). Processing included coil combination, phase-and frequency alignment, and eddy-current correction. For quality control, spectra were excluded if the averaged fitted spectrum had Cramer-Rao Lower Bounds (CRLBs)>5% for Glu or a tCr linewidth full-width-at-half-maximum (FWHM) > 19 Hz. Spectral quality metrics are provided in Supplemental Table S2. Acquisition and analysis parameters for fMRS were reported following the MRSinMRS checklist^38^ (Supplemental Table S3).

Our primary analysis focused on glutamate changes, and additionally changes in aspartate, glucose, and lactate are reported below. Other metabolites are reported in the Supplemental Materials. Metabolites with CRLBs exceeding 20% in more than 50% of participants were excluded from further analysis, and therefore GABA and glutamine (Gln) were not evaluated.

For the time series analysis, all data underwent dynamic fitting, enabling simultaneous fitting of a dynamic signal model to all transients. This method, also referred to as spectral-temporal or 2D fitting,^39^ reduces the number of free parameters by jointly modeling spectral and temporal information. By improving estimation of highly correlated model terms, dynamic fitting increases sensitivity to stimulus-induced changes in neurometabolite levels compared to traditional block averaging.^39,40^ It utilizes a design matrix to model the temporal response of each metabolite in the context of a GLM analysis. This matrix, combined with linear combination modeling of the spectral response at each timepoint, was fitted to the entire dataset concurrently using a least-squares approach. While the phase, shift, and baseline parameters were fixed across all timepoints, the *concentration* and *linewidth* (Gaussian linewidth, sigma) were constrained by the temporal model (i.e., design matrix). Notably, the linewidth was varied, considering previous findings suggesting that stimulus-induced changes in the BOLD effect could lead to line narrowing in MR spectra due to alterations in T2*.^8,41^ Other spectral fitting parameters are described in the Supplemental Materials. Design matrices followed a pseudo-block design, with one ~4-min baseline block (pre-medication) followed by eight ~4-min administration blocks, matching the timing and structure used in the phMRI analysis. (Supplemental Figure S1). In addition to the dynamic fitting results, we also show more ‘raw’ Glu timeseries, by extracting the estimates of initial (single transient) fits of Glu per subject, which were then averaged across subjects and smoothed using a moving average (bin size = 32) (Figure 1(a)).

### Statistics

#### phMRI and phMRS

Higher-level analyses investigated changes from baseline for each administration block (time-dependent effects) and condition differences in these changes from baseline per administration block (dose-dependent effects) for both the phMRS and phMRI data. Time-dependent effects were determined using one-sample *t*-tests, whereas dose-dependent effects were determined using paired *t*-tests. For phMRI, we used FSL Permutation-based Analysis of Linear Models^42^ (PALM; 5000 permutations, threshold-free cluster enhancement,^43^ family-wise error corrected *p*-values (p_FWE_) <0.05). P_FWE_ values were additionally corrected for multiple comparisons across the eight different assessed timepoints in PALM (i.e., eight administration-baseline contrast blocks). For dose-dependent analyses, we additionally applied a Bonferroni correction for three comparisons, yielding a significance threshold of p_FWE_ < 0.017. Proportion overlap between significant clusters and Harvard-Oxford structural atlas regions (cortical and subcortical) were determined using FSL’s *autoaq*. For phMRS, we used FSL’s *flameo*^35^ and performed multiple comparison correction for both time- (corrected for eight comparisons) and dose-dependent effects (corrected for 24 comparisons) using False Discovery Rate (FDR; stats package; R Core Team, 2013), with significance inferred when p_FDR_ < 0.05.

#### Association phMRI and receptor distribution maps

We conducted exploratory analyses to determine the association between the phMRI response maps (placebo vs S-ketamine contrast) and neurotransmitter receptor distributions. We investigated two timepoints which seemed to exemplify the observed rapid (block 2) and the slower, or more sustained, response (block 5) to S-ketamine and tested the associations with receptor distribution maps that ketamine has been shown to target,^16^ detailed in Table 1. To quantify these associations, we performed spin-tests^44,45^ in neuromaps^44^ (Supplemental Materials). Due to the exploratory nature of these analyses, we report uncorrected *p*-values.

**Table 1.** Overview of used PET maps to investigate the association between the phMRI response and receptor distributions.

Map	Source	Map	Source
Glutamate metabotropic receptor 5	Smart et al.^81^	NMDA receptor	Galovic et al.^82^
GABA_A_ receptor	Norgaard et al.^83^	Serotonin transporter	Beliveau et al.^84^
Acetylcholine transporter	Aghourian et al.^85^	Serotonin 2A receptor	Beliveau et al.^84^
Muscarinic M_1_ receptor	Naganawa et al.^86^	Dopamine transporter	Sasaki et al.^87^
Dopamine D_2_ receptor	Smith et al.^88^	Norepinephrine transporter	Ding et al.^89^
Opioid µ receptor	Turtonen et al.^90^	Opioid κ receptor	Vijay et al.^91^

GABA: gamma-aminobutyric acid; NMDA: N-methyl-D-aspartate; PET: positron emission tomography; phMRI: pharmacological magnetic resonance imaging.

#### Other statistical analyses

Subject-level BOLD contrast of parameter estimate (COPE) values (i.e., reflecting change from baseline for placebo, low dose, and high dose conditions) from phMRI analyses were extracted from the ACC phMRS voxel and analyzed using linear mixed-effects models (lme4 and lmerTest^46,47^) to determine both time- and dose-dependent effects (random intercept for participant to account for repeated measures, fitted using the maximum log-likelihood estimation). The influence of covariates (i.e., order of S-ketamine administration, mean FD, or %change in heart rate from baseline) was assessed to determine the stability of these findings. Model selection is detailed in the Supplemental Materials. Differences in blood plasma levels of S-ketamine and nor-S-ketamine between the S-ketamine conditions were analyzed using Wilcoxon signed rank tests. Subjective dissociation was analyzed using Friedman’s test on post-administration total CADSS scores, followed by Wilcoxon signed rank tests to determine dose-dependent differences (p_FDR_ < 0.05).

Bayesian multilevel modeling (package *brms*^48–50^; a high-level interface of *stan*^51^), was used to predict condition (placebo, low S-ketamine dose, high S-ketamine dose) based on individual-level COPE values. These regression models (four Markov chains, 2000 iterations, including 1000 warmup iterations) can account for the repeated measured structure of the data. Three different model types were specified: I. phMRI COPE values only, II. phMRS COPE values only, and III. COPE values from both phMRI and phMRS. Per model type, different models were specified (random intercept for participant, random intercept and slope per participant; see Supplemental Materials for additional information) and were compared using the Leave-One-Out Cross-Validation Information Criterion (LOO-IC) and the Widely Applicable Information Criterion (WAIC) to evaluate their predictive performance (*package loo*^52^). Per model type (I, II, III), the best-fitting model was selected, and model fit was once again compared using LOO-IC and WAIC. All Bayesian models were fitted on subjects with complete datasets for all predictors (*N* = 23).

All statistical analyses were conducted in RStudio v2022.02.1 (RStudio Team (2020); R version 4.3.1).

## Results

Of the 32 included participants (14 females; mean age = 22.8 years ± 5.4; age range = 19–43 years), 26 participants completed all 3 sessions. One subject was excluded from phMRI analyses due to insufficient data quality, resulting in *N* = 25 full datasets for dose-dependent phMRI analyses, and *N* = 27 placebo, *N* = 26 low dose, *N* = 29 high dose sessions were included for time-dependent phMRI analyses. For phMRS analyses, two subjects were removed after quality control, resulting in *N* = 24 full datasets for dose-dependent analyses, *N* = 26 placebo sessions, *N* = 25 low dose sessions, and *N* = 28 high dose sessions for time-dependent analyses. Details on participant inclusion and randomization is provided in Supplemental Table S1 and Figure S2. No differences in phMRI and phMRS data quality metrics (i.e., mean FD, CRLB_Glu_, FWHM_tCr)_ were observed between conditions (Supplemental Table S2). We observed no differences for blood-plasma concentrations of S-ketamine between the low and high dose, whereas we did observe significant differences for its main metabolite, nor-S-ketamine (V = 109; *p* = 0.003). This aligns with the known pharmacokinetics of S-ketamine, including rapid peak plasma levels, high clearance, and short elimination half-life.^53,54^ We report dose-dependent differences below, time-dependent changes are described in the Supplemental Materials.

### S-ketamine-induced phMRI response

**Figure 2.**
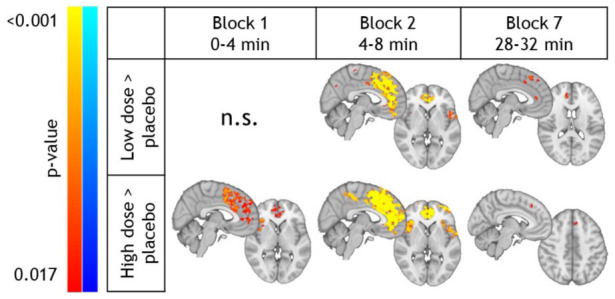
phMRI results: S-ketamine-induced effects on voxel-wise BOLD response. Clusters showing significantly higher BOLD changes (from baseline) compared to placebo for the low S-ketamine dose (top row) and high S-ketamine dose (bottom row). No significant voxel-wise differences were found for blocks 3, 4, 5 and 8. BOLD: blood-oxygen level dependent; phMRI: pharmacological magnetic resonance imaging; min: minutes.

At the voxel-wise level, both sub-anesthetic S-ketamine doses induced significant increases in BOLD signals, compared to placebo, across multiple time points (Figure 2). The spatial pattern of increased BOLD responses covers the medial frontal, (para)cingulate, and insular areas. While the phMRI response appeared less pronounced for the low compared to the high dose, these differences did not reach statistical significance. Additional maps and cluster information can be found in Supplemental Figure S3 and Supplemental Tables S4–S7.

PhMRI analyses within the phMRS voxel showed that both S-ketamine doses produced significantly greater BOLD responses than placebo, consistent with the voxel-wise analysis (time × dose interaction: *F*(14,621.6) = 1.83, *p* = 0.032; Figure 1(a)). Post-hoc comparisons showed this was the case for all infusion blocks. Furthermore, the change in BOLD was significantly larger in the high compared to the low dose 4–8 min after the start of the bolus administration (denoted as post-bolus in the following paragraphs). In the low S-ketamine dose, the change in BOLD in the first 4 min was significantly higher compared to 12–16 min post-bolus. In the high dose, the increase in BOLD in the first 4 min was significantly larger compared to 12–16 and 20–24 min post-bolus, and the increase in BOLD in 4–8 min post-bolus was significantly larger compared to all other timepoints (Supplemental Table S8). Sensitivity analyses indicated that these results were robust when testing covariates, such as change in heart rate (Supplemental Table S9), mean motion (FD, Supplemental Table S10), and order of S-ketamine administration (Supplemental Table S11). While mean motion explains some variance in BOLD signal changes after placebo or S-ketamine administration, adding FD as covariate does not significantly affect the dose- or block-dependent differences in the estimated marginal means of BOLD signal changes. This suggests that linear motion effects do not substantially confound the interpretation of the main findings, although more complex, non-linear motion-related contributions may still exist and should be considered when interpreting these results. Heart rate increased during the high S-ketamine dose compared to both low dose and placebo (time × dose interaction effect, *F*(14,595) = 2.51; *p* = 0.002; *post-hoc*: high > placebo 8–24 min and high>low 12–28 min post-bolus) (Supplemental Table S12). While heart rate changes were a significant predictor of BOLD signal alterations, post-hoc testing confirmed that the previously observed BOLD effects persisted even after correcting for these cardiovascular effects (Supplemental Table S9). Furthermore, change in HR and change in BOLD were not significantly associated during any of the infusion blocks (Supplemental Table S13).

While primarily employing the placebo condition as a control to compare against S-ketamine doses, we did observe BOLD signal fluctuations throughout the placebo imaging session, despite regressing out cerebrospinal fluid and white matter signals. We detected diminished BOLD signals across diffuse clusters, spanning temporo-parietal regions, the paracingulate gyrus, and precuneus (Supplemental Figure S3; Supplemental Table S4). These fluctuations might signify non-linear drifts unaccounted for by our nuisance regression or could potentially reflect changes in the participant’s cognitive state, such as a shift in attention during the session or feelings related to the absence of expected subjective effects.^55^ We conducted post-hoc analyses investigating differences between participants with prior (recreational) ketamine experience preceding the study and those without to examine this further and found that BOLD signals diminished significantly more in subjects with prior ketamine experience compared to those without at 4–8 min and 28–32 min post-bolus (Supplemental Figure S5; Supplemental Table S16).

### S-ketamine increases glutamate levels in the ACC

**Figure 3.**
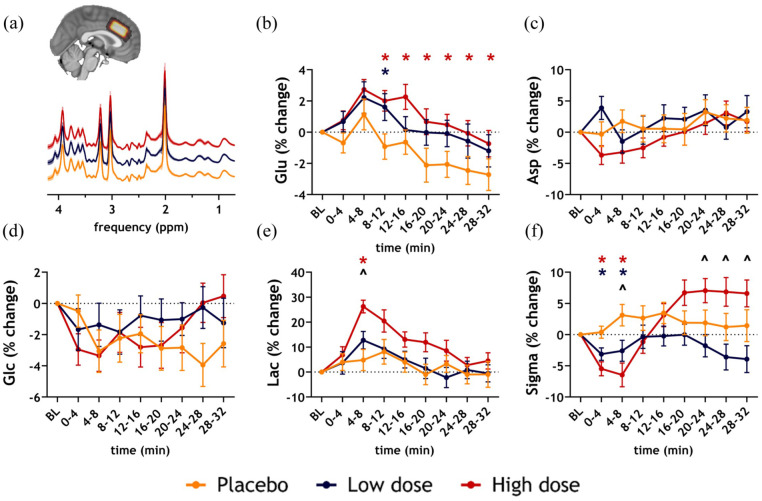
phMRS results: S-ketamine-induced changes in neurometabolite levels. (a) Mean spectra per condition across participants (mean + standard deviation). The insert shows the overlap of ACC MRS voxel across participants (lighter is more overlap) on a 1mm MNI brain. Mean percentage change + standard deviation from baseline per block for (b) glutamate (Glu) (c) aspartate (Asp) (d) glucose+tau (Glc) (e) lactate (Lac) (f) linewidth (Sigma). Differences between low dose and placebo conditions are illustrated with a blue asterisk; significant differences between high dose and placebo conditions are depicted with a red asterisk; significant differences between low and high dose conditions are depicted using a black circumflex. ACC: anterior cingulate cortex; BL: baseline; min: minute; MNI: Montreal Neurological Institute; MRS: magnetic resonance spectroscopy; ppm: parts per million.

Our results demonstrated that only the high dose of S-ketamine induced significantly higher Glu change (from baseline) compared to placebo from 8 min post-bolus until the end of the acquisition (block 3: *p*_FDR_ = 0.01, cohen’s *d* = 0.51; block 4: *p*_FDR_ = 0.01, cohen’s *d* = 0.54; block 5: *p*_FDR_ = 0.02, cohen’s *d* = 0.48; block 6: *p*_FDR_ = 0.05, cohen’s *d* = 0.58; block 7: *p*_FDR_ = 0.01, cohen’s *d* = 0.53; block 8: *p*_FDR_ = 0.02, cohen’s *d* = 0.46). The low dose increased Glu compared to placebo 8–12 min post-bolus (block 3: *p*_FDR_ = 0.04, cohen’s *d* = 0.49). We observed no significant differences between the low and high doses after multiple-comparison correction (Figure 1(a) and 3(b), Supplemental Tables S17–178. Glx (the composite of Glu and Gln) showed a similar response profile as Glu, but results did not reach statistical significance, potentially due to higher variance in the Gln estimates. (Supplemental Figure S6; Supplemental Tables S17–18).

No significant differences between placebo and S-ketamine conditions in Asp or Glc were found (Figure 3(c) and 3(d)). Contrastingly, Lac increased significantly more from baseline for the high S-ketamine dose in 4–8 min post-bolus compared to the low dose (*p*_FDR_ = 0.02, cohen’s *d* = 0.59) and placebo (*p*_FDR_ < 0.0001, cohen’s *d* = 0.94) (Figure 3(e)).

We observed that linewidth (sigma) mirrored the patterns observed in the phMRI analysis of the ACC voxel in the initial blocks after S-ketamine administration, namely significant decreases from baseline (reflecting increased BOLD signals) (Figure 3(f)). Linewidth decreased significantly in the high dose compared to the low dose (block 2: *p*_FDR_ = 0.045, cohen’s *d* = 0.46) and placebo (block 1: *p*_FDR_ < 0.005, cohen’s *d* = 0.78; block 2: *p*_FDR_ < 0.005, cohen’s *d* = 0.72). In the low dose, linewidth also decreased compared to placebo (block 1: *p*_FDR_ = 0.04, cohen’s *d* = 0.51; block 2: *p*_FDR_ = 0.045, cohen’s *d* = 0.45). In blocks 6–8, linewidth increased in the high compared to the low dose (block 6: *p*_FDR_ = 0.045, cohen’s *d* = 0.44; block 7: *p*_FDR_ = 0.04, cohen’s *d* = 0.48; block 8: *p*_FDR_ = 0.04, cohen’s *d* = 0.53). (Supplemental Tables S16–17).

### phMRI and phMRS provide complementary information in distinguishing S-ketamine doses and placebo

Next, we tested whether the received placebo or S-ketamine dose (condition) could be best predicted based on (i) the phMRI response (Supplemental Table S19), (ii) the phMRS response (Supplemental Table S20), or (iii) a combination of both. In the combined model, both phMRI and phMRS (Glu and Lac) predictors contributed to the best fit (Supplemental Table S21). Based on LOO-IC and the WAIC, the combined phMRI/phMRS model outperformed separate phMRI and phMRS models in predicting the received condition. The final model indicates that higher phMRI and higher Glu COPE values were associated with an increased probability of both S-ketamine dose conditions compared to placebo. Additionally, Lac COPE values were associated with an increased probability for the high S-ketamine condition compared to placebo. Predictors that distinguished between low and high S-ketamine doses were phMRI and Lac COPE values, as indicated by the difference in posterior distributions for these predictors between the low and high dose conditions (see Supplemental Table S21 for coefficients and difference scores).

### Dissociative experiences are associated with functional brain responses to S-ketamine

**Figure 4.**
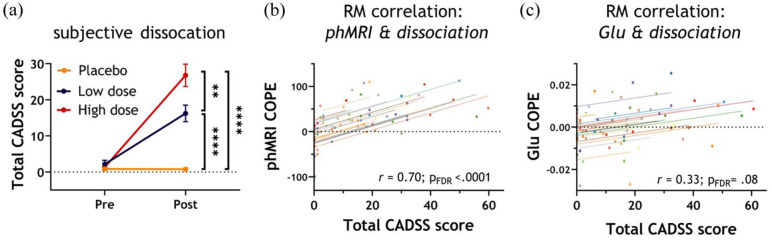
Subjective dissociation and its association with neuroimaging measures. (a). Total dissociation scores, as measured with the CADSS, before and after administration of placebo or S-ketamine. A dose-dependent increase in dissociation scores after administration was observed, with the high dose eliciting the highest dissociation in comparison to both low dose and placebo. **p < 0.01 **** *p* < 0.0001. (b) Repeated-measures association between phMRI COPE values and total CADSS scores at 4–8 min post-bolus. (c) Repeated-measures association between glutamate (Glu) COPE values and total CADSS scores at 0–4 min post-bolus. For the repeated measures correlations in graphs b and c, each colored line represents a different subject. These lines visually demonstrate how the overall correlation aligns with individual measurements from each subject. CADSS: Clinician-Administered Dissociative States Scale; COPE: contrast of parameter estimate; min: minutes; phMRI: pharmacological magnetic resonance imaging.

We observed that both S-ketamine doses induced a dissociative state in participants, with significantly stronger dissociation for the high compared to the low dose (χ^2^(2) = 41.6; *p* < 0.0001; *post-hoc* placebo vs low: *Z* = 300; *p* < 0.0001; placebo vs high: *Z* = 351; *p* < 0.0001; low vs high: *Z* = 278.5; *p* = 0.009; Figure 4(a)). Next, we examined whether the changes in the phMRI and phMRS responses were associated with dissociation scores. This revealed a significant and positive correlation between phMRI COPEs and total CADSS score in all administration blocks (Figure 4(b), Supplemental Table S22). The observed associations between Glu COPE and CADSS during administration blocks 1, 2, 3, and 6 did not survive correction for multiple comparisons (Figure 4C, Supplemental Table S23).

### S-ketamine phMRI response is associated with glutamatergic and opioid receptor distributions

**Figure 5.**
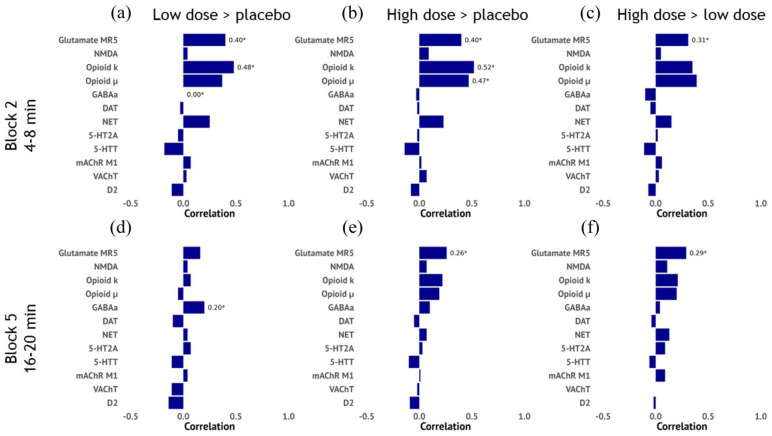
Associations between phMRI responses and receptor distribution maps. Horizontal bar plot showing the correlation between the phMRI contrast maps and receptor distribution maps based on positron emission tomography (PET) data from healthy volunteers. These data are plotted for the rapid response (block 2) for the (a). low > placebo, (b) high > placebo and (c) high > low contrasts and for the slower response (block 5) for the (d). low > placebo, (e) high > placebo and (f) high > low contrasts. Correlation coefficients were obtained using spin tests. phMRI: pharmacological magnetic resonance imaging; Glu MR5: glutamate metabotropic receptor 5; NMDA: N-methyl-D-aspartate receptor; GABAa: gamma-aminobutyric acid receptor A; 5-HTT: serotonin transporter; 5-HT2A: serotonin 2A receptor; VAChT: acetylcholine transporter; mAChR M_1_: metabotropic acetylcholine receptor 1; DAT: dopamine transporter; D2: dopamine D_2_ receptor; NET: norepinephrine transporter; min: minutes.

For the rapid response, we observed an association with the glutamate metabotropic receptor 5 (Glu MR5) and κ opioid receptor distributions for both S-ketamine doses (compared to placebo). For the high dose, the rapid response was additionally associated with the µ opioid receptor map. The sustained response was only associated with the Glu MR5 map for both S-ketamine conditions (Figure 5 and Supplemental Table S22).

## Discussion

The aim of our study was to evaluate an innovative MRI protocol capable of simultaneously assessing hemodynamic changes with phMRI and neurometabolism with phMRS to examine time- and dose-dependent responses to S-ketamine administration. This marks the first investigation employing a double-dose, placebo-controlled, within-subjects design with prolonged functional neuroimaging measurements. By integrating these two methods, we offer comprehensive and time-resolved insights into the functional dynamics induced by ketamine administration.

### phMRI response to ketamine

We observed a consistent increase in the BOLD response across various frontal, cingulate, and insular regions following both ketamine doses, aligning with findings from prior phMRI investigations involving intravenous ketamine challenges (for reviews, see McMillan R and Muthukumaraswamy^16^, Ionescu et al.^56^). However, our study stands out in several aspects of its design: we administered S-ketamine instead of racemic (R/S) ketamine, employed a double-dose rather than single-dose protocol, utilized a 7T-MRI scanner as opposed to lower field strengths, and extended our monitoring period to 30 min post-bolus, a departure from the more typical 10-min duration.

Ketamine phMRI investigations typically adopt a bolus with continuous infusion approach,^16^ although variations in administration schemes lead to differing plasma levels of ketamine. The S-ketamine dose used by Hoflich et al.,^61^ which closely resembles our low-dose infusion regimen, elicited significantly weaker and less widespread activation patterns compared to our results, despite using a signal model. These differences support the advantage of employing higher field strength acquisitions to enhance the SNR but may additionally be influenced by differences in analytical approaches.

Although we observed significant differences in the extent of dissociation between the low and high doses, these were not mirrored in the phMRI response. In the only other study that used multiple doses, the authors also did not report voxel-wise differences between their low and high subanesthetic doses of ketamine.^24^ These findings suggest that even relatively low subanesthetic doses can evoke substantial and widespread functional brain responses. In task-fMRI studies, low to moderate levels of sensory stimuli generally cause a linear increase in the BOLD response. However, the response can saturate, leading to a plateau.^62^ This has been observed across sensory modalities, including visual, auditory, and somatosensory systems. Similarly, such a plateau can be observed with ketamine administration. This is important to consider when interpreting phMRI results from strong pharmacological stimuli and emphasizes the importance of using complementary techniques that more directly reflect neuronal activation, such as phMRS.

S-ketamine’s systemic effects, particularly increased heart rate and blood pressure,^63^ can influence the BOLD response. While heart rate significantly increased at the high dose, including it in the model showed that BOLD changes remained consistent. The lack of a significant association between heart rate and BOLD alterations across infusion blocks further suggests that the observed phMRI effects are primarily driven by neurometabolic rather than cardiovascular changes.

Most studies examining the acute effects of ketamine have primarily measured the BOLD response over relatively short periods, providing limited insight into the prolonged response. Notably, Hoflich et al.^60,61^ conducted one of the few studies extending this observation window to approximately 45 min post-bolus infusion. However, their analysis approach for changes in BOLD signals solely incorporated the pharmacokinetic curve, potentially overlooking extended effects on the BOLD response. In contrast, our pseudo-block design facilitated evaluation of the phMRI response across multiple discrete bins. Intriguingly, following the peak response, we noted a transient signal decline to baseline, succeeded by a gradual BOLD signal increase in the high dose. While the origin of these temporal patterns remains speculative, evidence from electroencephalography (EEG) and magnetic encephalography (MEG) studies suggests the existence of multiple time courses of oscillatory activity with distinctive spatial profiles.^64^

When assessing the correlation between the phMRI response maps and receptor distributions, we observed that both the rapid and sustained functional responses to S-ketamine are tentatively associated with the glutamate MR5 receptor map, consistent with ketamine’s direct effect on glutamate release.^65^ In contrast, the lack of association with the NMDA receptor map may seem unexpected, given that NMDA receptors are ketamine’s primary pharmacological target. However, this finding is in line with earlier work,^66^ but may also be reflect limitations of the [^18^F]GE-179 tracer used to construct the NMDA receptor distribution, including its sensitivity to physiological factors, non-specific binding, and rapid metabolism.^67^ Furthermore, only the rapid response to S-ketamine appeared to be associated with κ and µ receptor maps. Interestingly, the interaction of ketamine with both µ and κ opioid receptors may be necessary for its antidepressant properties, as illustrated by the attenuation of ketamine’s antidepressant effect following (pre-)treatment with opioid receptor antagonists.^68,69^

### Glutamatergic contribution to phMRI changes: A role for phMRS

Pre-treatment studies with, for example, metabotropic glutamate 2/3 receptor agonists and lamotrigine, a voltage-gated sodium channel blocker, have demonstrated attenuation of the BOLD signal by these drugs, supporting the involvement of a ketamine-induced surge of glutamate in the cortical increases in BOLD signal seen following ketamine administration.^57,59^ Yet, to directly investigate neurometabolites involved in neuronal activity and neurotransmitter cycling, fMRS is currently the only non-invasive neuroimaging technique available. We recently reviewed the studies that investigated effects of acute ketamine on neurometabolite levels,^29^ and found quite mixed results. Although a few studies noted increased Glu, Gln or Glx in ACC regions,^31,58,70^ the majority of studies at either 3T or 7T did not report ketamine-induced changes therein. These inconsistencies may be related to differences in study design, the variation in voxel placement and other acquisition parameters, as well as analysis choices.

All prior ketamine MRS studies have analyzed neurometabolite levels as static signals (mostly at one time-point post-administration), providing a snapshot of neurometabolite levels following ketamine administration. However, this approach foregoes the dynamic nature of neurometabolite concentrations, as has repeatedly been shown in task-activation fMRS studies.^8,10,71^ As the first phMRS study taking a dynamic analysis approach, we could leverage its increased sensitivity to demonstrate time-dependent effects of ketamine administration on multiple neurometabolites. We found that particularly the high dose of S-ketamine increased glutamate up to ~3% from baseline between approximately 8–32 min following bolus administration, which reflects a different time course compared to the phMRI response, which seems to peak earlier. This suggests fundamental differences in the processes underlying changes in brain hemodynamics versus glutamate metabolism. In addition to glutamate, we also detect elevated lactate levels, up to ~25% from baseline, which have been linked to non-oxidative metabolism (glycolysis) and have been previously observed in task-related fMRS studies.^15^ This finding aligns with PET studies showing increased CBF and cerebral metabolic rate of glucose in the ACC, despite minimal changes in cerebral metabolic rate of oxygen,^72^ suggesting a shift toward glycolytic metabolism during the acute hyperglutamatergic state induced by ketamine. Beyond its metabolic role, lactate has also been implicated in neurovascular coupling through its vasodilatory properties^73^ and the expression of lactate receptors on blood-brain barrier cells.^74^ This dual involvement in both metabolism and hemodynamic regulation may underlie the observed parallels between lactate dynamics and the phMRI response.

Finally, we observed alterations in neurometabolites like tNAA and tCr, which are less commonly associated with neural activity changes. These alterations might be explained by changes in diffusion or neuronal microstructure^75,76^ or by incomplete correction for BOLD-induced effects on metabolite linewidths, as they represent the largest peaks in the spectrum.^8^ However, the exact nature of these changes and the role of dynamic fitting choices herein remains to be investigated.

### Subjective dissociation and anticipatory effects

Our finding that the intensity of dissociative experiences was positively associated with phMRI responses across all administration blocks suggests that the neural circuits engaged by S-ketamine’s functional effects are linked to the emergence of its dissociative phenomenology. Although exploratory, this relationship aligns with prior studies linking altered thalamocortical and fronto-parietal activity and connectivity to dissociative states under ketamine.^77–80^ The absence of consistent associations with Glu COPEs may reflect lower sensitivity of MRS to transient or spatially distributed glutamatergic changes underlying dissociation.

Our post-hoc analyses indicate that participants with prior ketamine experience exhibited greater reductions in BOLD signal during the placebo session. This may reflect altered expectancy or cognitive state in these individuals, potentially due to anticipation of drug effects or neurobiological adaptations from previous exposure. These findings have important implications for ketamine research and treatment. First, they highlight the need to consider prior drug experience as a potential confounder or moderator in both experimental and clinical studies. Second, they suggest that individual history with ketamine may shape both neural and subjective responses to treatment, which could influence efficacy, tolerability, or risk of adverse effects. However, we should note that some participants with no prior recreational exposure received one of the S-ketamine conditions before the placebo. Due to the limited sample size, we were not able to disentangle the effects of prior recreational use from potential influences of within-study exposure.

### Application and methodological considerations

Incorporating phMRS acquisitions into phMRI protocols combines the strengths of both techniques: the spatial resolution and SNR of fMRI acquisitions and the neural specificity of MRS. In addition to pre-treatment studies and receptor-informed fMRI, phMRS offers a unique opportunity to elucidate the neural mechanisms underlying the phMRI response. As such, combined phMRI/phMRS protocols add a valuable dimension to the existing neuroimaging toolbox, complementing established techniques such as EEG and [^18^F]FDG fPET/MRI, each providing distinct advantages in sensitivity, temporal resolution, spatial coverage, and specificity.

This integrated protocol can be readily adapted for future research in psychiatry and neurology. For example, our findings suggest it can identify neural signatures associated with individual differences in psychosis sensitivity — specifically, the propensity to experience dissociative or psychotomimetic symptoms following ketamine administration. Additionally, this approach holds promise for investigating mechanisms related to treatment response in conditions such as treatment-resistant depression. Furthermore, this protocol could be valuable to assess drug responses in populations with compromised neurovascular coupling, such as individuals with vascular pathology, where altered hemodynamic responses occur due to factors like vascular damage or arterial stiffness. In such cases, phMRS can assess the extent to which the neural response to medication remains intact. Moreover, the advantage of the simultaneous assessment of phMRI and phMRS is significant, as this reduces the risk of different outcomes due to habituation or sensitization that might occur if they were acquired on separate days. Additionally, our extended acquisition time enables the observation of effects over a longer time span compared to previous studies, potentially reflecting the activation of different receptors and downstream mechanisms.

We need to acknowledge several limitations of this study. Firstly, due to the significantly lower concentrations of neurometabolites compared to water, phMRS suffers from limited SNR in comparison to phMRI. However, our transition to 7T MRI has provided a substantial increase in SNR, enabling us to reliably estimate concentrations of several low concentration metabolites. Additionally, through the implementation of a dynamic shimming approach,^28^ we achieved good quality spectra and phMRI data with our interleaved protocol. Despite these improvements, we encountered challenges in dynamically fitting GABA and Gln resonances. Addressing this in future studies would be valuable, as these metabolites are closely linked to glutamate and could offer insights into the excitation-inhibition balance, potentially elucidating the phMRI results further. Furthermore, the temporal resolution of our interleaved acquisitions is predominantly dictated by the adiabatic pulses in the sLASER sequence, suggesting the need for future studies to explore methods for accelerating these acquisitions.

## Conclusion

Our study demonstrates a robust phMRI response in the dorsofrontal, cingulate, and insular cortices for both low and high subanesthetic doses of S-ketamine compared to placebo. The phMRI response patterns closely align with the distribution of glutamate and opioid receptors and are associated with subjective dissociation. Additionally, these hemodynamic changes coincide with increased glutamate and lactate levels in the ACC for the high S-ketamine dose. Together, simultaneous phMRI and phMRS measurements offer a more comprehensive understanding of the brain’s functional response to S-ketamine, as demonstrated by the improved accuracy in predicting S-ketamine doses and placebo conditions when combining the two techniques. Our novel interleaved phMRI-phMRS protocol bears considerable potential for concurrently assessing hemodynamic and neurometabolic effects of ketamine and other pharmacological agents. This innovative approach provides a pathway to disentangle the contributions of various neurometabolic processes to medication-induced functional brain changes. Moving forward, it holds great promise for enhancing our understanding of drug-brain interactions and facilitating a more direct investigation into the neurometabolic mechanisms underlying therapeutic response.

## Supplemental Material

sj-pdf-1-jcb-10.1177_0271678X251399023 – Supplemental material for Concurrent assessment of neurometabolism and brain hemodynamics to characterize the functional brain response to psychotropic drugs: an S-ketamine study Supplemental material, sj-pdf-1-jcb-10.1177_0271678X251399023 for Concurrent assessment of neurometabolism and brain hemodynamics to characterize the functional brain response to psychotropic drugs: an S-ketamine study by Daphne E Boucherie, Liesbeth Reneman, Jan Booij, Rogier Immink, Markus W Hollmann and Anouk Schrantee in Journal of Cerebral Blood Flow & Metabolism
