# Supplementary material for: Concurrent assessment of neurometabolism and brain hemodynamics to characterize the functional brain response to psychotropic drugs: an S-ketamine study
Source: J Cereb Blood Flow Metab. 2025 Nov 30:0271678X251399023. Online ahead of print. doi: 10.1177/0271678X251399023 (PMC12668993; doi:10.1177/0271678X251399023)
Supplement: sj-pdf-1-jcb-10.1177_0271678X251399023 – Supplemental material for Concurrent assessment of neurometabolism and brain hemodynamics to characterize the functional brain response to psychotropic drugs: an S-ketamine study [file sj-pdf-1-jcb-10.1177_0271678X251399023.pdf]

Supplementary Materials: “Concurrent assessment of neurometabolism and brain hemodynamics to characterize the functional brain response to psychotropic drugs: an S-ketamine study”

Supplementary Materials: “Concurrent assessment of neurometabolism and brain hemodynamics to characterize the functional brain response to psychotropic drugs: an S-ketamine study” 1
Supplementary Methods 3
Preprocessing of functional MRI data using fMRIprep 3
Anatomical data preprocessing 3
Functional data preprocessing 3
Design matrices for phMRI and phMRS analyses 4
Fig. S1. Design matrix for phMRI and phMRS 4
Fitting of phMRS spectra 5
Association between phMRI and receptor distribution maps 5
Model selection for Bayesian models to predict received dose 5
Supplementary Results 6
Participant inclusion and randomization 6
Fig. S2. Consort flow chart of participant inclusion. 6
Time-dependent changes in BOLD 6
Fig. S3. Time-dependent effects of S-ketamine on whole-brain phMRI BOLD 7
phMRI analyses using a signal model 8
Fig. S4. Analysis of phMRI data using a signal model analysis. 8
Exploratory analyses for diminished BOLD in the placebo condition 9
Dose-dependent changes in Glx 10
Time-dependent changes in neurometabolite levels 10
Supplementary Tables 11
Table S1. Subject randomization and included sessions per analysis. 11
Table S2. phMRI and phMRS data quality metrics. 11
Table S3. MRSinMRS acquisition parameters. 12

Table S4. Cluster information for time-dependent effects of S-ketamine on the phMRI signal for the placebo condition....113
Table S5. Cluster information for time-dependent effects of S-ketamine on the phMRI signal for the low S-ketamine dose condition....115
Table S6. Cluster information for time-dependent effects of S-ketamine on the phMRI signal for the high S-ketamine dose condition... 16
Table S7. Cluster information for dose-dependent effects of S-ketamine on the phMRI signal......18
Table S8. phMRI response: Linear mixed effect model for individual-level COPE values in the ACC MRS voxel. 20
Table S9. phMRI response and heart rate: Linear mixed effect model for individual-level COPE values in the ACC MRS voxel....22
Table S10. phMRI response and framewise displacement (FD): Linear mixed effect model for individual-level COPE values in the ACC MRS voxel....24
Table S11. phMRI response and medication order: Linear mixed effect model for individual-level COPE values in the ACC MRS voxel. A....26
Table S12. Heart rate: linear mixed effect model for %change in heart rate from baseline. 28
Table S13. Association between subject-level phMRI measures and heart rate....30
Table S14. Cluster information for time-dependent effects of S-ketamine on the phMRI signal using a signal model analysis....30
Table S15. Cluster information for dose-dependent effects of S-ketamine on the phMRI signal using a signal model analysis....31
Table S16. Cluster information for differences in the phMRI signal in the placebo condition for subjects who had prior experience with ketamine and those without....32
Table S17. phMRS: Time-dependent effects of S-ketamine on neurometabolite levels in the ACC. 33
Table S18. phMRS: Dose-dependent effects of S-ketamine on neurometabolite levels in the ACC. 37
Table S19. Bayesian multilevel modeling to estimate condition based on individual-level phMRI contrast of parameter estimates in the ACC 41
Table S20. Bayesian multilevel modeling to estimate condition based on individual-level phMRS contrast of parameter estimates in the ACC 42
Table S22. Association between subject-level neuroimaging measures and dissociation....44
Table S22. Associations between phMRI outcomes and receptor distribution maps 45
Supplementary references 47

Supplementary Methods

Preprocessing of functional MRI data using fMRIprep

Results included in this manuscript come from preprocessing performed using fMRIPrep 20.1.1[1] (RRID:SCR_016216), which is based on Nipype 1.5.0[2] (RRID:SCR_002502).

Anatomical data preprocessing

A total of 3 T1-weighted (T1w) images were found within the input BIDS dataset. All of them were corrected for intensity non-uniformity (INU) with N4BiasFieldCorrection[3], distributed with ANTs 2.2.0[4] (RRID:SCR_004757). The T1w-reference was then skull-stripped with a Nipype implementation of the antsBrainExtraction.sh workflow (from ANTs), using OASIS30ANTs as target template. Brain tissue segmentation of cerebrospinal fluid (CSF), white-matter (WM) and gray-matter (GM) was performed on the brain-extracted T1w using fast[5] (FSL 5.0.9, RRID:SCR_002823). A T1w-reference map was computed after registration of 3 T1w images (after INU-correction) using mri_robust_template[6] (FreeSurfer 6.0.1). Volume-based spatial normalization to two standard spaces (MNI152NLin2009cAsym, MNI152NLin6Asym) was performed through nonlinear registration with antsRegistration (ANTs 2.2.0), using brain-extracted versions of both T1w reference and the T1w template. The following templates were selected for spatial normalization: ICBM 152 Nonlinear Asymmetrical template version 2009c[7] [RRID:SCR_008796; TemplateFlow ID: MNI152NLin2009cAsym], FSL's MNI ICBM 152 non-linear 6th Generation Asymmetric Average Brain Stereotaxic Registration Model[8] [RRID:SCR_002823; TemplateFlow ID: MNI152NLin6Asym].

Functional data preprocessing

For each of the 3 BOLD runs found per subject (across all tasks and sessions), the following preprocessing was performed. First, a reference volume and its skull-stripped version were generated using a custom methodology of fMRIPrep. Head-motion parameters with respect to the BOLD reference (transformation matrices, and six corresponding rotation and translation parameters) are estimated before any spatiotemporal filtering using mcflirt[9] (FSL 5.0.9). A B0-nonuniformity map (or fieldmap) was estimated based on two (or more) echo-planar imaging (EPI) references with opposing phase-encoding directions, with 3dQwarp[10] (AFNI 20160207). Based on the estimated susceptibility distortion, a corrected EPI (echo-planar imaging) reference was calculated for a more accurate co-registration with the anatomical reference. The BOLD reference was then co-registered to the T1w reference using flirt[9] (FSL 5.0.9) with the boundary-based registration[11] cost-function. Co-registration was configured with nine degrees of freedom to account for distortions remaining in the BOLD reference. The BOLD time-series (including slice-timing correction when applied) were resampled onto their original, native space by applying a single, composite transform to correct for head-motion and susceptibility distortions. These resampled BOLD time-series will be referred to as preprocessed BOLD in original space, or just preprocessed BOLD. The BOLD time-series were resampled into standard space, generating a preprocessed BOLD run in MNI152NLin2009cAsym space. First, a reference volume and its skull-stripped version were generated using a custom methodology of fMRIPrep. Automatic removal of motion artifacts using independent component analysis[12] (ICA-AROMA) was applied to collect the “aggressive” noise-regressors and place them in the corresponding confounds file. Several confounding time-series were calculated based on the preprocessed BOLD: framewise displacement (FD), DVARS and three region-wise global signals. FD was computed using

two formulations following Power[13] (absolute sum of relative motions) and Jenkinson[14] (relative root mean square displacement between affines). FD and DVARS are calculated for each functional run, both using their implementations in Nipype (following the definitions by Power et al. 2014). The three global signals are extracted within the CSF, the WM, and the whole-brain masks. Additionally, a set of physiological regressors were extracted to allow for component-based noise correction[15] (CompCor).

Many internal operations of fMRIPrep use Nilearn 0.6.2[16] (RRID:SCR_001362), mostly within the functional processing workflow. For more details of the pipeline, see the section corresponding to workflows in fMRIPrep's documentation.

Following processing with fMRIprep, the first 14 volumes were discarded, and WM, CSF, and 6 motion parameters (i.e. rotation and translation in x, y, and z direction) and their first derivatives were regressed from the signal, followed by spatial smoothing (4mm FWHM).

Design matrices for phMRI and phMRS analyses

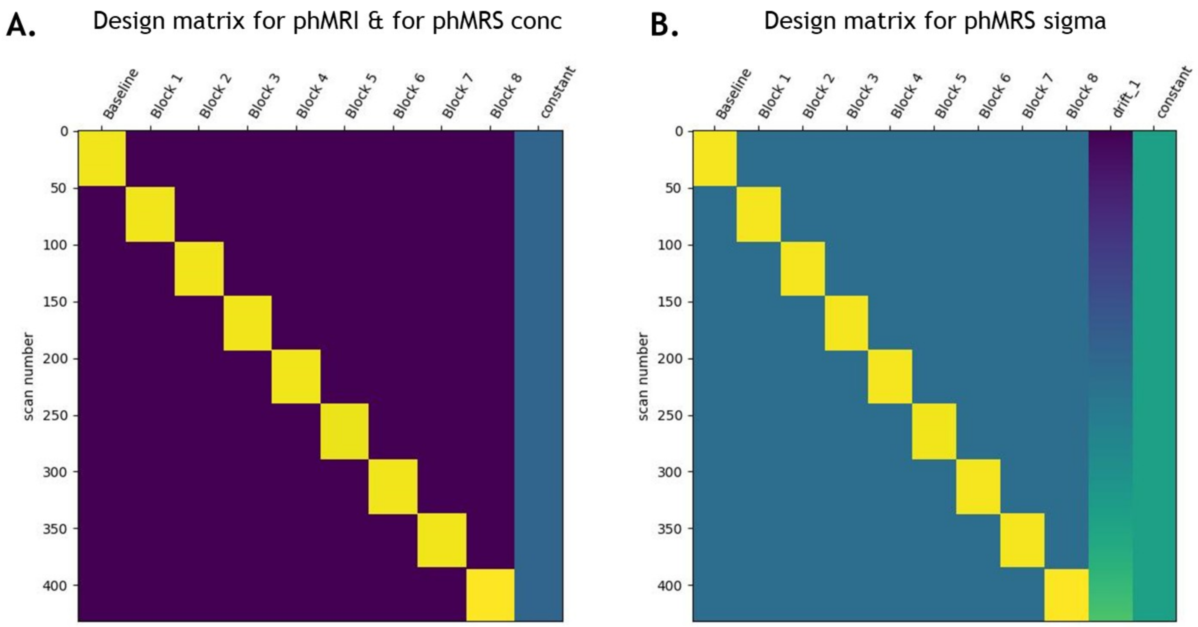

Fig. S1. Design matrix for phMRI and phMRS. A. The design matrix used for the first-level phMRI analysis and for the *concentration* parameter in the first-level phMRS analysis. **B.** The design matrix used for the *sigma* parameter in the first-level phMRS analysis.

conc: concentration; phMRI: pharmacological magnetic resonance imaging; phMRS: pharmacological magnetic resonance spectroscopy.

Fitting of phMRS spectra

For the mean phMRS spectra, the fitting process involved aligning basis spectra to the complex-valued spectrum in the frequency domain. A basis set was simulated based on our MRS sequence in FSL-MRS. The basis set was simulated using full pulse descriptions and 60 spatially resolved points in each dimension[17, 18]. The basis set comprised 19 metabolites and a measured macromolecular baseline, available at https://github.com/mrshub/mm-consensus-data-collection[19]. A Voigt lineshape model, incorporating one Gaussian and one Lorentzian line broadening parameter, was applied to shift and broaden the basis spectra. Metabolite basis spectra shared the same lineshape model and parameters, except for macromolecules, which had distinct parameters. Specifically, the lineshape model employed four linewidth parameters: one Lorentzian and one Gaussian parameter for all metabolites, and another set for the macromolecular (MM) basis spectrum. The Lorentzian parameter was enforced to be positive, as was the constant term of the Gaussian broadening. Additionally, a complex second-order polynomial baseline was concurrently fitted. Model fitting utilized the truncated Newton algorithm from scipy[20].

For the dynamic fitting, design matrices were created using nilearn[16], and incorporated separate regressors for each of the blocks, similar to the phMRI analysis, as well as a baseline regressor. The design matrix fitted to the *concentration* and *linewidth* were the same, except that a linear drift parameter was added to the *linewidth* design matrix to reflect potential drift in linewidth as a result of BOLD drift. Note that we applied the design matrix to the Gaussian line-broadening parameter (denoted as sigma), whereas the Lorentzian parameter remained fixed across time.

Association between phMRI and receptor distribution maps

Receptor distribution maps (obtained from publicly available positron emission tomography (PET) maps) were obtained from neuromaps[21], an open source toolbox accessing, transforming and analyzing structural and functional brain annotations. All receptor maps of interest were available in neuromaps, with the exception of the NMDA (N-methyl-D-aspartate) receptor map. All maps were resampled to 2mm isotropic resolution and parcellated into 250 atlas regions, based on the Cammoun atlas available in neuromaps[22]. To quantify the association between phMRI response maps and various receptor distributions, we performed a spin-test[21, 23], a statistical method employed to compute null models by randomly rotating data, allowing for the assessment of statistical significance between two spatial maps[24]. Spatial null distributions were generated for each of the investigated placebo-drug-contrast based on whole-brain t-stat maps by applying the approach by Burt et al.[25] (default parameters as provided by the brainSMASH software; 1000 permutations) and were subsequently correlated to each of the parcellated receptor maps. Due to the exploratory nature of these analyses, we report the uncorrected p-values per association.

Model selection for Bayesian models to predict received dose

Model selection for all Bayesian models was conducted as follows: the most complicated model in terms of main predictors and interaction effects was tested initially, and subsequently predictors not significantly contributing to the prediction were removed from the model. The final model included the remaining significant main and interaction effects, and main effects of predictors in the significant interaction effects.

Supplementary Results

Participant inclusion and randomization

In total, 32 participants were included and randomized into one of six possible medication orders. Subject inclusion and randomization is detailed in Fig. S2 and Table S1.

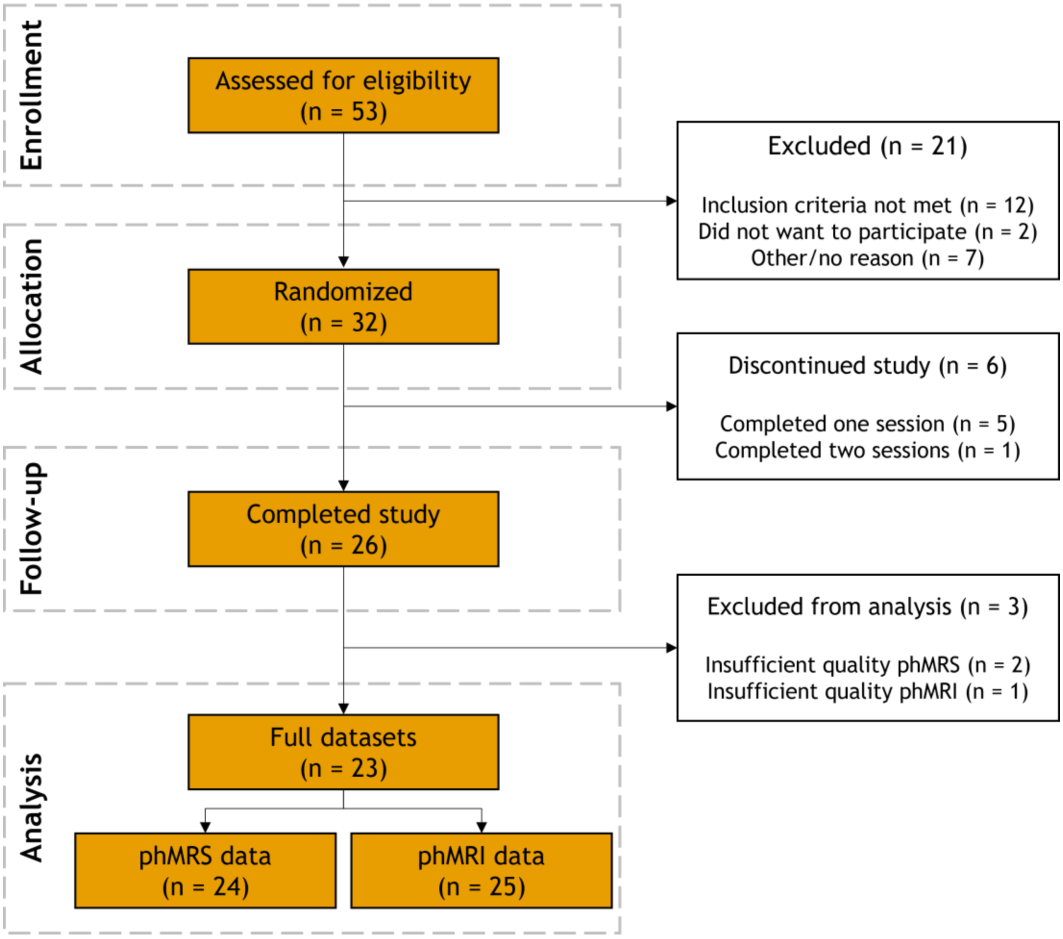

Fig. S2. Consort flow chart of participant inclusion. In total, N=26 subjects completed all sessions. Per condition, N=27 subjects completed the placebo session, N=26 the low dose session, and N=29 participants completed the high dose sessions.

phMRI: pharmacological magnetic resonance imaging; phMRS: pharmacological magnetic resonance spectroscopy.

Time-dependent changes in BOLD

At the voxel-wise level, both sub-anesthetic S-ketamine doses induced significant increases in the BOLD signals, compared to baseline, across multiple time points (Supplementary Figure S2). The spatial pattern of increased BOLD responses covers the medial frontal, (para)cingulate, and insular areas. The response to both S-ketamine doses was characterized by a strong early rise in the BOLD signal (0-8 minutes of administration), which subsequently normalized to baseline, but increased again after 16 minutes for the high S-ketamine dose only. Cluster information for these changes is provided in Supplementary Tables S3-S5.

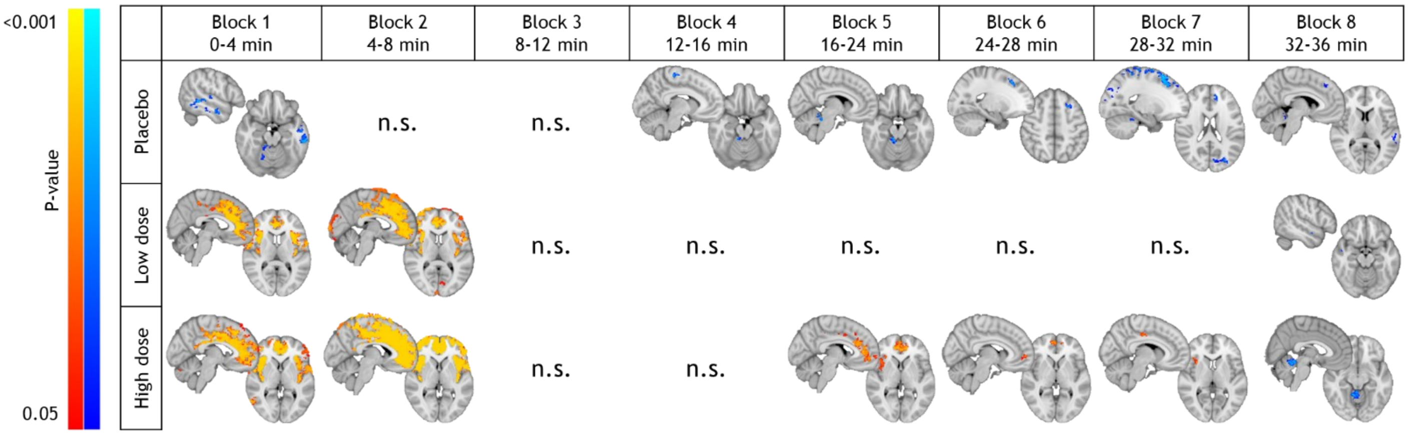

Fig. S3. Time-dependent effects of S-ketamine on whole-brain phMRI BOLD. Clusters from the voxel-based analysis showing significant changes from baseline for each condition (placebo, low dose of S-ketamine, and high dose of S-ketamine) per block.

BOLD: blood-oxygen level dependent; phMRI: pharmacological magnetic resonance imaging.

phMRI analyses using a signal model

Repeating our analysis using a signal model (gamma variate function; Supplementary Figure S3A) replicated the strong phMRI responses reported in prior studies (Supplementary Figure S3B). Cluster information for these effects is provided in Supplementary Tables S13-S14.

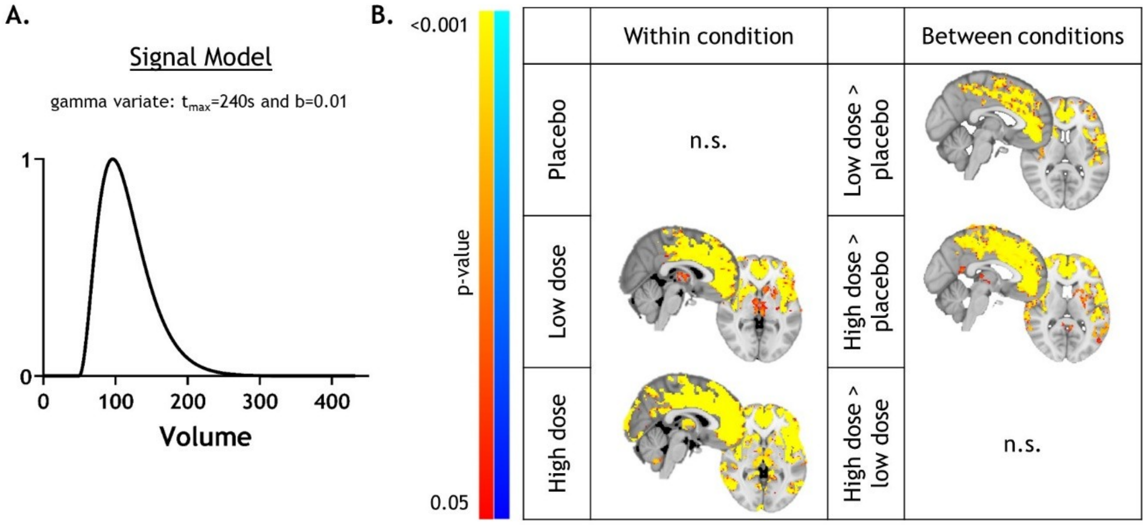

Fig. S4. Analysis of phMRI data using a signal model analysis. A. The used signal model regressor. We modeled this regressor as a gamma variate using t_{max}=240s and b=0.01. B. Main effects of the signal model regressor per condition are depicted on the left (within condition). Dose-dependent effects are visualized on the right (between conditions).

phMRI: pharmacological magnetic resonance imaging.

Exploratory analyses for diminished BOLD in the placebo condition

As one possible explanation for the observed clusters showing decreased BOLD in the placebo condition, we hypothesized that participants may experience a release of uncertainty whether S-ketamine was given. We therefore conducted exploratory analyses to investigate differences between participants with prior ketamine experience (N=14) and those without (N=13) and found that BOLD signals diminished significantly more in subjects with prior ketamine experience compared to those without at 4-8 minutes and 24-28 minutes post-placebo administration (see Figure S5 and Supplementary Table S15 for cluster information).

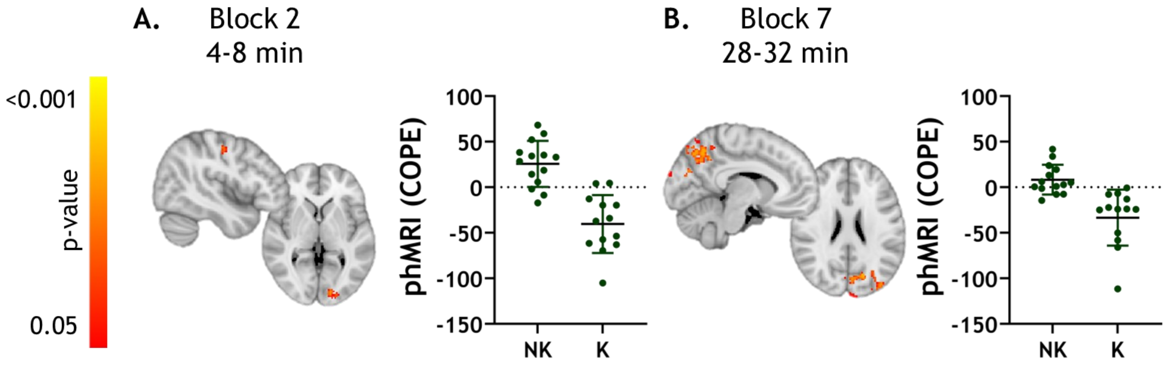

Fig. S5. Effect of prior ketamine use on placebo response. Group differences between subjects who had experienced ketamine prior to the experiments and those who had not in the placebo session. **A.** Significant differences between the two groups during infusion block 2. **B.** Significant differences between the two groups during infusion block 7.

COPE = contrast of parameter estimates; K= prior ketamine experience; NK= no prior ketamine experience; phMRI: pharmacological magnetic resonance imaging.

Dose-dependent changes in Glx

To evaluate the overlap between changes in Glu and Glx, we analyzed Glx using the same approach as Glx. We observed no significant differences in Glx between placebo, low dose, and high dose of S-ketamine for any of the investigated timepoints.

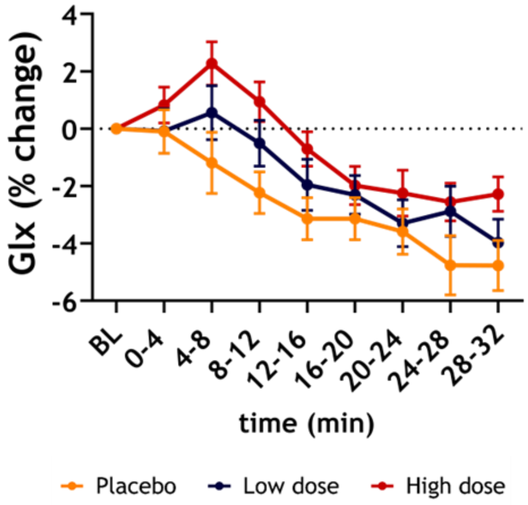

Fig. S6. phMRS results: S-ketamine-induced changes in Glx levels. Percentage change + standard deviation from baseline per block for glutamate + glutamine (Glx) Differences between low dose and placebo conditions are illustrated with a blue asterisk; significant differences between high dose and placebo conditions are depicted with a red asterisk; significant differences between low and high dose conditions are depicted using a black circumflex.

BL: baseline; min: minute; MRS: magnetic resonance spectroscopy.

Time-dependent changes in neurometabolite levels

Compared to baseline, Glu significantly increased during the second (p_{FDR}=0.001), third (p_{FDR}=0.01), and fourth (p_{FDR}=0.01) administration blocks (4-16 minutes post-administration), respectively. No significant changes in Glu were found for the low dose compared to baseline. We observed no significant changes from baseline for aspartate for any of the conditions. For glucose, a significant decrease in glucose from baseline was observed during the high S–ketamine dose only (block 1: p_{FDR}=0.01; block 2: p_{FDR}=0.01; block 4: p_{FDR}=0.048). Compared to baseline, lactate increased 4-12 min post-administration in the low dose (block 2: p_{FDR}=0.005; block 3: p_{FDR}=0.048) and in the first 28 minutes post-administration for the high S-ketamine dose (block 1: p_{FDR}=0.03; block 2: p_{FDR}<0.0001; block 3: p_{FDR}=0.002; block 4: p_{FDR}=0.003; block 5: p_{FDR}=0.004; block 6: p_{FDR}=0.03; block 7: p_{FDR}=0.02).

Gaussian lineshape parameter sigma decreased during the first administration block in the low condition (p_{FDR}=0.02) and during the first two post-administration blocks in the high condition (block 1: p_{FDR}<0.0001; block 2: p_{FDR}=0.0003), in line with the phMRI findings in the ACC voxel.

Time- and dose-dependent changes in other neurometabolites are more difficult to interpret, as they are not as explicitly involved in the neurometabolic cycle or have not been extensively assessed in fMRS studies previously. For example, we observe changes in PE, Ins, tNAA, tCr, and tCh. PE increased significantly in the low dose compared to placebo, whereas Ins increased significantly in the high dose compared to placebo. Additionally, significant differences in tNAA, tCr and tCh between the conditions were observed (Supplementary Tables S13-14).

Supplementary Tables

Table S1. Subject randomization and included sessions per analysis.

P: placebo session; L: low dose session; H: high dose session

A. Study **randomization**						
	P-L-H	P-H-L	L-H-P	L-P-H	H-P-L	H-L-P
Subjects randomized	5	5	5	7	6	7
Subjects discontinuing study	0	1	0	1	2	2

B. Included sessions per condition			
	Placebo	Low dose	High dose
Completed all sessions	26	26	26
Completed one session	1	0	3
Completed two sessions	-	1	1
Total time-dependent analyses	27	27	30

Table S2. phMRI and phMRS data quality metrics. Per condition, the full-width at half maximum (FWHM; in Hz) of total creatine (tCr) (N=24), the Cramér-Rao lower bounds (CRLB) of glutamate (Glu) (N=24), and the mean framewise displacement (FD; in mm) (N=25) after scrubbing are provided as well as results from a repeated measures ANOVA to determine dose-dependent differences in these quality metrics. Per quality metric, the mean and standard deviation are provided.

ANOVA: analysis of variance; phMRI: pharmacological magnetic resonance imaging; phMRS: pharmacological magnetic resonance spectroscopy.

	Placebo	Low dose	High dose	Statistics	
				F(df)	*p*
$FWHM_{tCr}$	13.21 (2.2)	14.06 (2.0)	13.51 (1.51)	F(2,46) = 1.44	0.25
$CRLB_{Glu}$	2.77 (0.27)	2.80 (0.32)	2.92 (0.34)	F(2,46) = 2.30	0.11
Mean FD	0.29 (0.19)	0.26 (0.13)	0.28 (0.14)	F(2,48) = 0.67	0.46

Table S3. MRSinMRS acquisition parameters.

	sLASER
**1. Hardware**	
a. Field Strength	7T
b. Manufacturer	Philips
c. Model	Achieva
d. RF coil	Quadrature birdcage transmit and 32-channel phased-array receive coils (Nova Medical)
e. Additional hardware	N/A
**2. Acquisition**	
a. Pulse sequence	sLASER (Interleaved with) 3D-EPI
b. Volume of interest	Dorsal ACC
c. Nominal VOI size	30 x 20 x 15 mm
d. Repetition time (TR), echo time (TE)	TR/TE=3500/36 ms
e. Total number of acquisitions/averages	448
f. Additional sequence parameters	3000 Hz; 1024 data points
g. Water suppression method	VAPOR
h. Shimming method	HOS-DLT (Boer et al. 2020)
i. Triggering or motion correction method	None
**3. Data analysis methods and outputs**	
a. Analysis software	FSL-MRS v.2.1.13
b. Processing steps deviating from quoted product	None
c. Output measure	Dynamic fitting on unscaled spectra
d. Quantification references and assumptions	Basis set generated for this sequence (Schrantee et al. 2023)
**4. Data quality**	
a. Reported variables	FWHM, CRLB
b. Data exclusion criteria	Metabolites not evaluated when >50% subjects >20% CRLB; Spectra were excluded when CRLB Glu>5% or tCr FWHM >19Hz.
c Quality measures of postprocessing model fitting	Glu CRLB, tCr FWHM
d. Sample spectrum	Figure 3A

Table S4. Cluster information for time-dependent effects of S-ketamine on the phMRI signal for the placebo condition. Only clusters with a minimal size of 10 voxels and with proportion overlap of at least 5 with an atlas region are shown. The Harvard-Oxford structural atlas (cortical and subcortical) was used for identification and clusters were extracted using autoaq in FSL.

phMRI: pharmacological magnetic resonance imaging.

Block	Cluster	# voxels	p-value	Overlap	Region
1	7	502	0.016	14.05	Posterior Superior Temporal Gyrus
				9.87	Posterior Middle Temporal Gyrus
				7.70	Middle Temporal Gyrus
				7.59	Anterior Middle Temporal Gyrus
	6	177	0.026	7.87	Brain-Stem
				7.08	Lingual Gyrus
	5	160	0.017	42.86	Posterior Middle Temporal Gyrus
				13.52	Posterior Inferior Temporal Gyrus
	3	21	0.041	32.19	Planum Temporale
				17.29	Heschl's Gyrus (includes H1 and H2)
4	5	45	0.033	26.82	Precentral Gyrus
	4	16	0.034	28.55	Brain-Stem
5	1	78	0.023	35.32	Brain-Stem
6	5	140	0.024	26.64	Superior Frontal Gyrus
				20.09	Middle Frontal Gyrus
	4	51	0.028	30.14	Superior Frontal Gyrus
				11.33	Paracingulate Gyrus
	3	15	0.042	28.00	Superior Frontal Gyrus
				14.13	Paracingulate Gyrus
				12.07	Juxtapositional Lobule Cortex
	2	10	0.045	58.40	Middle Frontal Gyrus
7	24	2575	0.004	15.11	Superior Frontal Gyrus
				11.52	Middle Frontal Gyrus
				7.88	Precentral Gyrus
				5.13	Postcentral Gyrus
	23	508	0.021	9.76	Temporal Occipital Fusiform Cortex
				6.53	Posterior Parahippocampal Gyrus
				5.68	Lingual Gyrus
	22	374	0.02	25.41	Lateral Occipital Cortex
				15.68	Cuneal Cortex
				8.37	Precuneous Cortex
	21	128	0.033	49.98	Postcentral Gyrus
				17.70	Precentral Gyrus
	20	103	0.024	30.60	Precentral Gyrus
				11.07	Postcentral Gyrus
	19	81	0.033	53.41	Lateral Occipital Cortex
	18	37	0.029	50.49	Postcentral Gyrus
	17	35	0.04	69.03	Precuneous Cortex
				5.14	Postcentral Gyrus
	15	16	0.046	34.63	Occipital Pole
				10.31	Lateral Occipital Cortex
	14	14	0.044	40.14	Posterior Temporal Fusiform Cortex

				14.00	Temporal Occipital Fusiform Cortex
	12	11	0.043	17.82	Anterior Superior Temporal Gyrus
				14.82	Planum Polare
				7.55	Anterior Middle Temporal Gyrus
	11	10	0.039	25.20	Precuneous Cortex
				17.40	Posterior Cingulate Gyrus
8	6	82	0.019	8.55	Brain-Stem
	5	60	0.04	26.72	Planum Temporale
				23.33	Posterior Superior Temporal Gyrus
	4	17	0.047	17.88	Planum Temporale
				14.06	Heschl's Gyrus (includes H1 and H2)
				12.00	Anterior Superior Temporal Gyrus
				11.29	Posterior Superior Temporal Gyrus
	3	15	0.043	33.67	Superior Frontal Gyrus
				12.80	Paracingulate Gyrus

Table S5. Cluster information for time-dependent effects of S-ketamine on the phMRI signal for the low S-ketamine dose condition. Only clusters with a minimal size of 10 voxels and with proportion overlap of at least 5 with an atlas region are shown. The Harvard-Oxford structural atlas (cortical and subcortical) was used for identification and clusters were extracted using autoaq in FSL.

phMRI: pharmacological magnetic resonance imaging.

Block	Cluster	# voxels	p-value	Overlap	Region
1	9	3896	>0.001	28.58	Anterior Cingulate Gyrus
				22.88	Paracingulate Gyrus
				6.57	Frontal Pole
				5.15	Frontal Medial Cortex
	8	3463	>0.001	10.07	Frontal Pole
				9.70	Insular Cortex
				6.61	Inferior Frontal Gyrus
				5.16	Precentral Gyrus
	7	2578	>0.001	15.01	Insular Cortex
				10.40	Central Opercular Cortex
				6.09	Parietal Operculum Cortex
				5.40	Anterior Supramarginal Gyrus
				5.26	Frontal Operculum Cortex
				5.18	Precentral Gyrus
	6	107	0.025	42.72	Frontal Pole
				30.62	Frontal Orbital Cortex
	5	14	0.043	37.64	Posterior Cingulate Gyrus
				31.21	Precentral Gyrus
				9.50	Precuneous Cortex
2	10	17416	>0.001	11.11	Frontal Pole
				8.64	Paracingulate Gyrus
				8.01	Anterior Cingulate Gyrus
	9	7918	0.003	6.12	Precentral Gyrus
				5.64	Lateral Occipital Cortex
	8	650	0.012	25.13	Occipital Pole
				7.72	Cuneal Cortex
	7	166	0.03	6.28	Occipital Pole
				5.43	Lateral Occipital Cortex
				5.23	Occipital Fusiform Gyrus
	6	101	0.02	50.73	Frontal Pole
					Frontal Orbital Cortex
	5	57	0.036	65.67	Lateral Occipital Cortex
	4	22	0.041	34.91	Lateral Occipital Cortex
					Lateral Occipital Cortex
					Middle Temporal Gyrus
	3	10	0.046	37.90	Precentral Gyrus
					Postcentral Gyrus

Table S6. Cluster information for time-dependent effects of S-ketamine on the phMRI signal for the high S-ketamine dose condition. Only clusters with a minimal size of 10 voxels and with proportion overlap of at least 5 with an atlas region are shown. The Harvard-Oxford structural atlas (cortical and subcortical) was used for identification and clusters were extracted using autoaq in FSL.
phMRI: pharmacological magnetic resonance imaging.

Block	Cluster	# voxels	p-value	Overlap	Region
1	15	18591	>0.001	10.61	Frontal Pole
				8.65	Anterior Cingulate Gyrus
				8.08	Paracingulate Gyrus
	14	6393	0.001	8.22	Frontal Pole
				6.02	Central Opercular Cortex
				6.00	Precentral Gyrus
				5.86	Insular Cortex
				5.61	Middle Frontal Gyrus
	12	80	0.033	38.83	Superior Parietal Lobule
				5.50	Postcentral Gyrus
	11	74	0.038	32.59	Superior Frontal Gyrus
				13.51	Frontal Pole
	10	23	0.04	38.83	Postcentral Gyrus
				24.57	Superior Parietal Lobule
	9	21	0.042	28.67	Occipital Pole
	8	16	0.047	45.13	Angular Gyrus
				5.38	Lateral Occipital Cortex
	7	11	0.048	34.91	Lateral Occipital Cortex
				15.73	Superior Parietal Lobule
2	3	38228	>0.001	12.74	Frontal Pole
				5.48	Middle Frontal Gyrus
				5.01	Paracingulate Gyrus
5	8	1333	0.01	33.15	Paracingulate Gyrus
				30.15	Anterior Cingulate Gyrus
				7.16	Frontal Pole
				6.42	Frontal Medial Cortex
	7	344	0.015	31.29	Insular Cortex
				17.08	Frontal Operculum Cortex
				6.49	Central Opercular Cortex
				6.39	Frontal Orbital Cortex
	6	69	0.04	35.77	Anterior Cingulate Gyrus
				33.96	Paracingulate Gyrus
	5	65	0.037	30.69	Posterior Cingulate Gyrus
				11.51	Precentral Gyrus
				9.37	Precuneous Cortex
	4	45	0.041	42.18	Anterior Cingulate Gyrus
				11.51	Juxtapositional Lobule Cortex
				11.24	Paracingulate Gyrus
	3	10	0.045	69.80	Anterior Cingulate Gyrus
				10.00	Juxtapositional Lobule Cortex
				8.50	Posterior Cingulate Gyrus
6	2	108	0.028	43.51	Paracingulate Gyrus

				22.66	Frontal Pole
				14.79	Frontal Medial Cortex
7	12	133	0.034	37.83	Posterior Cingulate Gyrus
				27.62	Anterior Cingulate Gyrus
				6.36	Juxtapositional Lobule Cortex
	11	115	0.027	32.55	Insular Cortex
				24.83	Frontal Operculum Cortex
				5.50	Frontal Orbital Cortex
	10	52	0.03	60.50	Frontal Pole
	9	47	0.038	28.68	Inferior Frontal Gyrus
				20.85	Inferior Frontal Gyrus
				6.36	Middle Frontal Gyrus
	8	13	0.044	48.31	Posterior Cingulate Gyrus
				6.00	Precentral Gyrus

Table S7. Cluster information for dose-dependent effects of S-ketamine on the phMRI signal. Only clusters with a minimal size of 10 voxels and with proportion overlap of at least 5 with an atlas region are shown. The Harvard-Oxford structural atlas (cortical and subcortical) was used for identification and clusters were extracted using autoaq in FSL.

phMRI: pharmacological magnetic resonance imaging.

Low dose > placebo					
Block	Cluster	# voxels	p-value	Overlap	Region
2	18	3594	>0.001	32.06	Paracingulate Gyrus
				24.09	Anterior Cingulate Gyrus
				8.32	Superior Frontal Gyrus
	17	3072	0.001	11.03	Middle Frontal Gyrus
				10.68	Precentral Gyrus
				7.42	Postcentral Gyrus
				6.18	Anterior Supramarginal Gyrus
				5.92	Posterior Supramarginal Gyrus
				5.26	Inferior Frontal Gyrus
	16	339	0.006	18.80	Precentral Gyrus
				18.04	Middle Frontal Gyrus
				16.55	Inferior Frontal Gyrus
	15	193	0.004	46.63	Precuneous Cortex
				13.83	Postcentral Gyrus
	14	145	0.004	28.92	Frontal Orbital Cortex
				15.23	Frontal Operculum Cortex
				5.81	Inferior Frontal Gyrus
				5.46	Insular Cortex
	13	102	0.008	37.23	Precuneous Cortex
				14.15	Cuneal Cortex
	12	48	0.011	46.69	Lateral Occipital Cortex
	11	37	0.012	35.05	Insular Cortex
				18.16	Frontal Operculum Cortex
	10	23	0.011	21.43	Superior Parietal Lobule
				16.22	Lateral Occipital Cortex
7	6	50	0.01	49.52	Anterior Cingulate Gyrus
				27.10	Paracingulate Gyrus
	5	33	0.01	51.27	Superior Frontal Gyrus
				13.79	Paracingulate Gyrus
	4	19	0.012	59.63	Paracingulate Gyrus
				23.37	Anterior Cingulate Gyrus
**High dose > placebo**					
Block	Cluster	# voxels	p-value	Overlap (%)	Region
1	25	2063	0.004	27.84	Paracingulate Gyrus
				20.30	Anterior Cingulate Gyrus
				11.17	Superior Frontal Gyrus
	24	407	0.004	21.47	Frontal Operculum Cortex
				15.69	Insular Cortex
				8.45	Central Opercular Cortex
	23	404	0.01	24.10	Precentral Gyrus
				14.40	Inferior Frontal Gyrus

				10.80	Middle Frontal Gyrus
	22	295	0.01	30.74	Precentral Gyrus
				19.56	Middle Frontal Gyrus
				8.14	Inferior Frontal Gyrus
	21	120	0.01	28.55	Superior Frontal Gyrus
				21.02	Middle Frontal Gyrus
	20	44	0.012	48.66	Anterior Supramarginal Gyrus
				8.07	Parietal Operculum Cortex
				5.59	Planum Temporale
	19	25	0.012	71.76	Central Opercular Cortex
	18	22	0.013	31.09	Postcentral Gyrus
				27.23	Central Opercular Cortex
				7.68	Planum Temporale
				5.32	Parietal Operculum Cortex
				5.05	Anterior Supramarginal Gyrus
	17	12	0.016	63.75	Frontal Pole
				7.83	Paracingulate Gyrus
	16	12	0.016	54.25	Anterior Cingulate Gyrus
	15	11	0.016	35.27	Middle Frontal Gyrus
				17.73	Superior Frontal Gyrus
2	7	14528	>0.001	11.50	Paracingulate Gyrus
				11.34	Frontal Pole
				8.96	Anterior Cingulate Gyrus
				5.50	Superior Frontal Gyrus
				5.28	Middle Frontal Gyrus
	6	2083	0.001	12.68	Inferior Frontal Gyrus
				8.69	Frontal Operculum Cortex
				8.00	Insular Cortex
				7.39	Frontal Orbital Cortex
				6.82	Middle Frontal Gyrus
				6.77	Precentral Gyrus
	5	96	0.012	40.89	Superior Frontal Gyrus
	4	52	0.012	38.19	Frontal Orbital Cortex
				9.77	Insular Cortex
	3	10	0.014	31.30	Inferior Frontal Gyrus
				15.40	Middle Frontal Gyrus
				5.70	Inferior Frontal Gyrus
7	1	16	0.013	26.00	Superior Frontal Gyrus
				24.75	Paracingulate Gyrus

Table S8. phMRI response: Linear mixed effect model for individual-level COPE values in the ACC MRS voxel. A. Bayesian Information Criterion (BIC)=6501. All p-values are corrected using False Discovery Rate (FDR) multiple comparison correction. **B.** Time-dependent effects are reported, i.e. differences between time points within a single condition. **C.** Dose-dependent differences are reported, i.e. differences between conditions per time point. Significant values ($p_{FDR}<0.05$) are highlighted in bold.

Model: COPE_fMRI ~ condition * block + (1 | Subject)

ACC: anterior cingulate cortex; COPE: contrast of parameter estimate; MRS; magnetic resonance spectroscopy; phMRI: pharmacological magnetic resonance imaging.

A. Linear mixed effects model results		
condition	F(df)=93 .61(2,637.53)	**p<.0001**
block	F(df)=5.04(7,621.55)	**p<.0001**
condition * block	F(df)=1.82(14,621.55)	**p=.032**

B. Post hoc analysis: time-dependent effects

	Block 1 : Block 2	Block 1 : Block 3	Block 1 : Block 4	Block 1 : Block 5	Block 1 : Block 6	Block 1 : Block 7	Block 1 : Block 8	Block 2 : Block 3
placebo	p=.73	p=.84	p=.73	p=.99	p=.73	p=.73	p=.84	p=.77
low	p=.77	**p=.05**	**p=.05**	p=.17	p=.09	p=.17	p=.09	p=.09
high	p=.45	**p=.002**	**p=.01**	p=.08	**p=.03**	p=.07	p=.07	**P<.0001**

	Block 2 : Block 4	Block 2 : Block 5	Block 2 : Block 6	Block 2 : Block 7	Block 2 : Block 8	Block 3 : Block 4	Block 3 : Block 5	Block 3 : Block 6
placebo	p=.87	p=.77	p=.84	p=.84	p=.84	p=.84	p=.84	p=.84
low	p=.09	p=.42	p=.20	p=.42	p=.21	p=.91	p=.55	p=.77
high	**p=.0006**	**p=.004**	**p=.002**	**p=.004**	**p=.004**	p=.60	p=.21	p=.38

	Block 3 : Block 7	Block 3 : Block 8	Block 4 : Block 5	Block 4 : Block 6	Block 4 : Block 7	Block 4 : Block 8	Block 5 : Block 6	Block 5 : Block 7
placebo	p=.84	p=.88	p=.73	p=.99	p=.97	p=.84	p=.73	p=.79
3low	p=.55	p=.77	p=.64	p=.77	p=.64	p=.77	p=.77	p=.97
high	p=.23	p=.23	p=.60	p=.84	p=.63	p=.63	p=.84	p=.96

	Block 5 : Block 8	Block 6 : Block 7	Block 6 : Block 8	Block 7 : Block 8
placebo	p=.84	p=.97	p=.84	p=.88

low	p=.77	p=.77	p=.97	p=.77
high	p=.96	p=.84	p=.84	p=.99

C. Post hoc analysis: dose-dependent effects

	Block 1	Block 2	Block 3	Block 4	Block 5	Block 6	Block 7	Block 8
placebo: low	**p<.0001**	**p<.0001**	**p=.05**	**p=.003**	**p=.011**	**p=.0007**	**p=.0002**	**p=.003**
placebo: high	**p<.0001**	**p<.0001**	**p=.05**	**p=.0002**	**p=.002**	**p=.0001**	**p<.0001**	**p=.0001**
low:high	p=.49	**p=.02**	p=.85	p=.42	p=.50	p=.47	p=.60	p=.32

Table S9. phMRI response and heart rate: Linear mixed effect model for individual-level COPE values in the ACC MRS voxel. A. Bayesian Information Criterium (BIC)=5039 All p-values are corrected using False Discovery Rate (FDR) multiple comparison correction. **B.** Time-dependent effects are reported, i.e. differences between time points within a single condition. **C.** Dose-dependent differences are reported, i.e. differences between conditions per time point. Significant values (p_{FDR}<0.05) are highlighted in bold.

Model: COPE_fMRI ~ condition * block + HR_change + (1 | Subject)

ACC: anterior cingulate cortex; COPE: contrast of parameter estimate; MRS; magnetic resonance spectroscopy; phMRI: pharmacological magnetic resonance imaging.

A. Linear mixed effects model results		
condition	F(df)=85.63(2,614.8)	**p<.0001**
block	F(df)=4.94(7, 596.4)	**p<.0001**
%change in heart rate	F(df)=1.17(1,624.1)	p=.28
condition * block	F(df)=1.81(14, 595.8)	**p=.035**

B. Post hoc analysis: time-dependent effects

	Block 1 : Block 2	Block 1 : Block 3	Block 1 : Block 4	Block 1 : Block 5	Block 1 : Block 6	Block 1 : Block 7	Block 1 : Block 8	Block 2 : Block 3
placebo	p=.87	p=.88	p=.87	p=.97	p=.87	p=.87	p=.88	p=.87
low	p=.80	**p=.04**	**p=.04**	p=.16	p=.08	p=.08	**p=.04**	p=.08
high	p=.38	**p=.003**	**p=.02**	p=.1	**p=.04**	p=.07	p=.07	**p<.0001**

	Block 2 : Block 4	Block 2 : Block 5	Block 2 : Block 6	Block 2 : Block 7	Block 2 : Block 8	Block 3 : Block 4	Block 3 : Block 5	Block 3 : Block 6
placebo	p=.88	p=.87	p=.88	p=.88	p=.88	p=.88	p=.88	p=.88
low	p=.08	p=.41	**p=.018**	p=.21	p=.08	p=.98	p=.65	p=.80
high	**p=.0006**	**p=.003**	**p=.002**	**p=.003**	**p=.003**	p=.69	p=.33	p=.54

	Block 3 : Block 7	Block 3 : Block 8	Block 4 : Block 5	Block 4 : Block 6	Block 4 : Block 7	Block 4 : Block 8	Block 5 : Block 6	Block 5 : Block 7
placebo	p=.88	p=.88	p=.87	p=.97	p=.97	p=.95	p=.87	p=.87
low	p=.80	p=.98	p=.65	p=.80	p=.80	p=.98	p=.80	p=.80
high	p=.38	p=.38	p=.69	p=.87	p=.76	p=.76	p=.83	p=.91

	Block 5 : Block 8	Block 6 : Block 7	Block 6 : Block 8	Block 7 : Block 8
placebo	p=.88	p=.97	p=.97	p=.96
low	p=.65	p=.98	p=.80	p=.80
high	p=.91	p=.87	p=.87	p>.99

C. Post hoc analysis: dose-dependent effects								
	Block 1	Block 2	Block 3	Block 4	Block 5	Block 6	Block 7	Block 8
placebo: low	**p<.0001**	**p<.0001**	p=.04	**p=.006**	**p=.011**	**p=.002**	**p=.001**	**p=.014**
placebo: high	**p<.0001**	**p<.0001**	p=.04	**p=.0003**	**p=.002**	**p=.0002**	**p<.0001**	**p=.0001**
low:high	p=.61	p=.02	p=.87	p=.37	p=.57	p=.50	p=.40	p=.16

Table S10. phMRI response and framewise displacement (FD): Linear mixed effect model for individual-level COPE values in the ACC MRS voxel. A. Bayesian Information Criterium (BIC)=6577. All p-values are corrected using False Discovery Rate (FDR) multiple comparison correction. **B.** Time-dependent effects are reported, i.e. differences between time points within a single condition. **C.** Dose-dependent differences are reported, i.e. differences between conditions per time point. Significant values (p_{FDR}<0.05) are highlighted in bold.
Model:
COPE_fMRI ~ condition * block + FD * condition + (1 | Subject)

ACC: anterior cingulate cortex; COPE: contrast of parameter estimate; MRS; magnetic resonance spectroscopy; phMRI: pharmacological magnetic resonance imaging.

A. Linear mixed effects model results		
condition	F(df)=2.51(2,632.7)	p=.082
block	F(df)=5.51(7,619.9)	**p<.0001**
FD	F(df)=32.72(1,520.9)	**p<.0001**
condition * block	F(df)=1.95(14,619.9)	**p=.019**
condition * FD	F(df)=16.40(2,597.7)	**p<.0001**

B. Post hoc analysis: time-dependent effects

	Block 1 : Block 2	Block 1 : Block 3	Block 1 : Block 4	Block 1 : Block 5	Block 1 : Block 6	Block 1 : Block 7	Block 1 : Block 8	Block 2 : Block 3
placebo	p=.69	p=.83	p=.69	p=.99	p=.69	p=.69	p=.83	p=.69
low	p=.76	**p=.037**	**p=.037**	p=.15	p=.07	p=.15	p=.07	p=.07
high	p=.52	**p=.0007**	**p=.0063**	p=.05	**p=.02**	**p=.04**	**p=.04**	**p<.0001**

	Block 2 : Block 4	Block 2 : Block 5	Block 2 : Block 6	Block 2 : Block 7	Block 2 : Block 8	Block 3 : Block 4	Block 3 : Block 5	Block 3 : Block 6
placebo	p=.83	p=.69	p=.83	p=.83	p=.83	p=.83	p=.83	p=.83
low	p=.07	p=.34	p=.15	p=.34	p=.16	p=.91	p=.52	p=.76
high	**p=.0004**	**p=.003**	**p=.001**	**p=.003**	**p=.003**	p=.58	p=.19	p=.36

	Block 3 : Block 7	Block 3 : Block 8	Block 4 : Block 5	Block 4 : Block 6	Block 4 : Block 7	Block 4 : Block 8	Block 5 : Block 6	Block 5 : Block 7
placebo	p=.83	p=.83	p=.69	p=.99	p=.97	p=.83	p=.69	p=.69
low	p=.52	p=.76	p=.62	p=.76	p=.62	p=.76	p=.76	p=.97
high	p=.21	p=.21	p=.58	p=.83	p=.62	p=.62	p=.83	p=.95

	Block 5 : Block 8	Block 6 : Block 7	Block 6 : Block 8	Block 7 : Block 8
placebo	p=.83	p=.97	p=.83	p=.88

low	p=.76	p=.76	p=.97	p=.76				
high	p=.95	p=.83	p=.83	p=.99				

C. Post hoc analysis: dose-dependent effects

	Block 1	Block 2	Block 3	Block 4	Block 5	Block 6	Block 7	Block 8
placebo:low	**p<.0001**	**p<.0001**	**p<.0001**	**p<.0001**	**p<.0001**	**p<.0001**	**p<.0001**	**p<.0001**
placebo:high	**p<.0001**	**p<.0001**	**p<.0001**	**p<.0001**	**p<.0001**	**p<.0001**	**p<.0001**	**p<.0001**
low:high	p=.42	p=.42	p=.42	p=.42	p=.42	p=.42	p=.42	p=.42

Table S11. phMRI response and medication order: Linear mixed effect model for individual-level COPE values in the ACC MRS voxel. **A.** Bayesian Information Criterium (BIC)=6372. All p-values are corrected using False Discovery Rate (FDR) multiple comparison correction. **B.** Time-dependent effects are reported, i.e. differences between time points within a single condition. **C.** Dose-dependent differences are reported, i.e. differences between conditions per time point. Significant values (p_{FDR}<0.05) are highlighted in bold.

Model: COPE_fMRI ~ condition * block + order_med + (1 | Subject)

ACC: anterior cingulate cortex; COPE: contrast of parameter estimate; MRS; magnetic resonance spectroscopy; phMRI: pharmacological magnetic resonance imaging.

A. Linear mixed effects model results		
condition	F(df)=92.91(2, 638.4)	**p<.0001**
block	F(df)=5.05(7, 622.1)	**p<.0001**
medication order	F(df)=0.47(1,29)	p=.79
condition * block	F(df)=1.82(14, 602)	**p=.032**

B. Post hoc analysis: time-dependent effects

	Block 1 : Block 2	Block 1 : Block 3	Block 1 : Block 4	Block 1 : Block 5	Block 1 : Block 6	Block 1 : Block 7	Block 1 : Block 8	Block 2 : Block 3
placebo	p=.73	p=.84	p=.73	p=.99	p=.73	p=.73	p=.84	p=.73
low	p=.77	**p=.048**	**p=.048**	p=.17	p=.09	p=.17	p=.09	p=.09
high	p=.45	**p=.002**	**p=.01**	p=.08	**p=.03**	p=.07	p=.07	**p<.0001**

	Block 2 : Block 4	Block 2 : Block 5	Block 2 : Block 6	Block 2 : Block 7	Block 2 : Block 8	Block 3 : Block 4	Block 3 : Block 5	Block 3 : Block 6
placebo	p=.84	p=.73	p=.84	p=.84	p=.84	p=.84	p=.84	p=.84
low	p=.11	p=.42	p=.21	p=.42	p=.21	p=.91	p=.55	p=.82
high	**p=.0006**	**p=.004**	**p=.002**	**p=.004**	**p=.004**	p=.60	p=.21	p=.38

	Block 3 : Block 7	Block 3 : Block 8	Block 4 : Block 5	Block 4 : Block 6	Block 4 : Block 7	Block 4 : Block 8	Block 5 : Block 6	Block 5 : Block 7
placebo	p=.84	p=.88	p=.73	p=.99	p=.97	p=.84	p=.73	p=.73
low	p=.59	p=.82	p=.67	p=.82	p=.67	p=.82	p=.82	p=.97
high	p=.23	p=.23	p=.60	p=.84	p=.63	p=.63	p=.84	p=.96

	Block 5 : Block 8	Block 6 : Block 7	Block 6 : Block 8	Block 7 : Block 8
placebo	p=.84	p=.97	p=.84	p=.88
low	p=.82	p=.82	p=.97	p=.82

high	p=.96	p=.84	p=.84	p=.99				
C. Post hoc analysis: dose-dependent effects								
	Block 1	Block 2	Block 3	Block 4	Block 5	Block 6	Block 7	Block 8
placebo: low	**p<.0001**	**p<.0001**	p=.050	**p=.003**	**p=.012**	**p=.0007**	**p=.0002**	**p=.003**
placebo: high	**p<.0001**	**p<.0001**	p=.050	**p=.0003**	**p=.002**	**p=.0001**	**p<.0001**	**p=.0001**
low:high	p=.49	**p=.02**	p=.86	p=.43	p=.50	p=.47	p=.61	p=.32

Table S12. Heart rate: linear mixed effect model for %change in heart rate from baseline. **A.** Bayesian Information Criterium (BIC)=5039. All p-values are corrected using False Discovery Rate (FDR) multiple comparison correction. **B.** Time-dependent effects are reported, i.e. differences between time points within a single condition. **C.** Dose-dependent differences are reported, i.e. differences between conditions per time point. Significant values (p_{FDR}<0.05) are highlighted in bold. Model: HR_change ~ condition * block + (1 | Subject)

A. Linear mixed effects model results		
condition	F(df)=34.38(2,634.7)	**p<.0001**
block	F(df)=8.30(7,618.9)	**p<.0001**
condition * block	F(df)=2.30(14,618.9)	**p=.004**

B. Post hoc analysis: time-dependent effects

	Block 1 : Block 2	Block 1 : Block 3	Block 1 : Block 4	Block 1 : Block 5	Block 1 : Block 6	Block 1 : Block 7	Block 1 : Block 8	Block 2 : Block 3
placebo	p=.94	p=.94	p=.94	p=.94	p=.98	p=.94	p=.94	p=.94
low	p=.14	p=.24	p=.92	p=.72	p=.23	p=.14	p=.14	p=.72
high	**p=.0003**	**p<.0001**	**p=.0007**	p=.08	p=.16	p=.37	p=.75	p=.24

	Block 2 : Block 4	Block 2 : Block 5	Block 2 : Block 6	Block 2 : Block 7	Block 2 : Block 8	Block 3 : Block 4	Block 3 : Block 5	Block 3 : Block 6
placebo	p=.94	p=.94	p=.94	p=.94	p=.94	p=.98	p=.94	p=.94
low	p=.14	p=.06	**p=.006**	**p=.002**	**p=.002**	p=.23	p=.14	**p=.018**
high	p=.75	p=.07	**p=.027**	**p=.006**	**p=.0007**	p=.14	**p=.002**	**p=.0006**

	Block 3 : Block 7	Block 3 : Block 8	Block 4 : Block 5	Block 4 : Block 6	Block 4 : Block 7	Block 4 : Block 8	Block 5 : Block 6	Block 5 : Block 7
placebo	p=.98	p=.94	p=.94	p=.94	p=.98	p=.94	p=.94	p=.94
low	**p=.006**	**p=.006**	p=.75	p=.24	p=.14	p=.14	p=.43	p=.24
high	**P=.0001**	**p<.0001**	p=.13	p=.07	**p=.017**	**p=.002**	p=.75	p=.42

	Block 5 : Block 8	Block 6 : Block 7	Block 6 : Block 8	Block 7 : Block 8
placebo	p=.98	p=.95	p=.94	p=.94
low	p=.24	p=.74	p=.74	p=.97
high	p=.14	p=.65	p=.26	p=.53

C. Post hoc analysis: dose-dependent effects

	Block 1	Block 2	Block 3	Block 4	Block 5	Block 6	Block 7	Block 8
placebo:	p=.99	p=.24	p=.08	p=.81	p=.645	p=.13	p=.14	p=.24

low								
placebo: high	p=.99	**p=.004**	**p<.0001**	**p=.0001**	**p=.007**	p=.13	p=.17	p=.24
low:high	p=.99	p=.06	**p-=.0002**	**p=.0003**	**p=.019**	**p=.005**	**p=.007**	p=.052

Table S13. Association between subject-level phMRI measures and heart rate. A. Per infusion block, the results from the repeated measures correlations between the phMRI COPE values and %change in heart rate are provided.

COPE: contrast of parameter estimates; phMRI: pharmacological magnetic resonance imaging

	Block 1 (0 - 4 min)		Block 2 (4 – 8 min)		Block 3 (8 – 12 min)		Block 4 (12 – 16 min)	
	r	p_{FDR}	r	p_{FDR}	r	p_{FDR}	r	p_{FDR}
**phMRI**	-.17	.71	-.21	.71	-.01	.94	-.06	.90
	Block 5 (16 – 20 min)		Block 6 (20 – 24 min)		Block 7 (24 – 28 min)		Block 8 (28 – 32 min)	
	r	p_{FDR}	r	p_{FDR}	r	p_{FDR}	r	p_{FDR}
**phMRI**	.-.04	.90	.09	.90	-.07	..90	.17	.71

Table S14. Cluster information for time-dependent effects of S-ketamine on the phMRI signal using a signal model analysis. Only clusters with a minimal size of 10 voxels and with proportion overlap of at least 5 with an atlas region are shown. The Harvard-Oxford structural atlas (cortical and subcortical) was used for identification and clusters were extracted using autoaq in FSL.

phMRI: pharmacological magnetic resonance imaging.

Cluster	# voxels	p-value	Overlap	Region
Low S-ketamine dose				
1	50369	>0.001	9.0022	Frontal Pole
1	50369	>0.001	5.4227	Precentral Gyrus
High S-ketamine dose				
1	87346	>0.001	7.283	Frontal Pole

Table S15. Cluster information for dose-dependent effects of S-ketamine on the phMRI signal using a signal model analysis. Only clusters with a minimal size of 10 voxels and with proportion overlap of at least 5 with an atlas region are shown. The Harvard-Oxford structural atlas (cortical and subcortical) was used for identification and clusters were extracted using autoaq in FSL.

phMRI: pharmacological magnetic resonance imaging.

Cluster	# voxels	p-value	Overlap	Region
Placebo vs low S-ketamine dose				
5	29027	>0.001	7.3794	Frontal Pole
			6.0104	Precentral Gyrus
			5.8954	Paracingulate Gyrus
			5.3096	Anterior Cingulate Gyrus
4	31	0.013	33.4194	Precuneous Cortex
			42.25	Posterior Middle Temporal Gyrus
			10.4643	Posterior Inferior Temporal Gyrus
Placebo vs high S-ketamine dose				
10	48161	>0.001	7.578	Frontal Pole
9	199	0.012	30.8894	Lingual Gyrus
			5.5879	Temporal Occipital Fusiform Cortex
8	103	0.013	47.0688	Right Thalamus
			33.8806	Left Thalamus
7	55	0.008	50.7455	Posterior Cingulate Gyrus
			13.5455	Precuneous Cortex
6	37	0.01	43.6486	Posterior Cingulate Gyrus
			16.3243	Precuneous Cortex

Table S16. Cluster information for differences in the phMRI signal in the placebo condition for subjects who had prior experience with ketamine and those without. Only clusters with a minimal size of 10 voxels and with proportion overlap of at least 5 with an atlas region are shown. The Harvard-Oxford structural atlas (cortical and subcortical) was used for identification and clusters were extracted using autoaq in FSL.

phMRI: pharmacological magnetic resonance imaging

Block	Cluster	# voxels	p-value	Overlap	Region
2	15	112	0.03	28.00	Occipital Pole
				6.15	Intracalcarine Cortex
	14	62	0.036	37.76	Postcentral Gyrus
				29.66	Precentral Gyrus
	13	29	0.042	23.41	Supracalcarine Cortex
				16.14	Intracalcarine Cortex
				14.45	Cuneal Cortex
	12	11	0.045	26.55	Cuneal Cortex
				6.00	Lateral Occipital Cortex
7	7	1708	0.015	27.03	Precuneous Cortex
				16.22	Lateral Occipital Cortex
				5.50	Cuneal Cortex
	6	159	0.018	35.11	Postcentral Gyrus
					Precentral Gyrus
					Anterior Supramarginal Gyrus
	5	54	0.035	36.72	Occipital Pole
					Cuneal Cortex
	4	41	0.043	38.24	Lateral Occipital Cortex
					Occipital Pole

Table S17. phMRS: Time-dependent effects of S-ketamine on neurometabolite levels in the ACC. Per condition (placebo, low dose of S-ketamine, and high dose of S-ketamine), and per infusion block, the % change from baseline with standard deviation (SD) are provided, as well as the original p-values and the false discovery rate (FDR)-corrected p-values (p_{FDR}).

ACC: anterior cingulate cortex; phMRS: pharmacological magnetic resonance spectroscopy.

Placebo																
	Block 1				Block 2				Block 3				Block 4			
	%	±SD	p	p_{FDR}	%	±SD	p	p_{FDR}	%	±SD	p	p_{FDR}	%	±SD	p	p_{FDR}
Asc	-0.27	4.38	0.48	0.480	5.21	4.61	0.13	0.360	3.77	6.03	0.27	0.390	2.03	5.01	0.34	0.390
Asp	-0.33	1.98	0.44	0.440	1.74	1.82	0.17	0.370	0.55	2.13	0.40	0.440	0.53	2.24	0.41	0.440
tCr	-0.25	0.32	0.22	0.250	0.34	0.51	0.26	0.260	-0.45	0.52	0.20	0.250	-0.64	0.64	0.17	0.250
GSH	0.19	1.36	0.44	0.440	4.11	1.34	0.004	0.010	2.61	1.57	0.05	0.070	3.83	1.31	0.004	0.010
Glu	-0.70	0.64	0.14	0.170	1.13	0.99	0.13	0.170	-0.92	0.82	0.13	0.170	-0.64	0.78	0.21	0.210
Glx	-0.10	0.76	0.49	0.448	-1.19	1.07	0.14	0.159	-2.23	0.73	0.003	0.003	-3.14	0.73	0.0001	0.0002
Ins	0.13	0.36	0.36	0.410	0.19	0.42	0.33	0.410	-0.37	0.76	0.31	0.410	-0.32	0.49	0.26	0.410
Lac	3.95	3.36	0.13	0.270	4.80	4.24	0.13	0.270	8.14	4.97	0.06	0.270	3.98	4.07	0.17	0.270
tNAA	-0.13	0.24	0.29	0.290	-0.18	0.24	0.23	0.290	-0.16	0.26	0.28	0.290	-0.43	0.39	0.14	0.220
tCh	0.66	0.68	0.17	0.370	0.78	0.72	0.14	0.370	0.86	0.75	0.13	0.370	0.47	0.79	0.28	0.370
PE	-2.02	1.82	0.14	0.140	-4.08	1.28	0.002	0.002	-6.17	1.79	0.001	0.002	-6.18	1.46	0.0001	0.0004
Scyllo	-2.18	2.90	0.23	0.380	-0.84	2.92	0.39	0.390	-2.96	3.18	0.18	0.390	-6.35	2.92	0.02	0.160
Glc+Tau	-0.50	1.04	0.32	0.320	-3.06	1.36	0.02	0.060	-2.24	1.54	0.08	0.090	-1.94	1.22	0.06	0.080
sigma	0.38	0.97	0.35	0.350	3.12	1.73	0.04	0.170	2.64	1.96	0.10	0.250	3.47	1.71	0.03	0.170
	Block 5				Block 6				Block 7				Block 8			
	%	±SD	p	p_{FDR}	%	±SD	p	p_{FDR}	%	±SD	p	p_{FDR}	%	±SD	p	p_{FDR}
Asc	3.00	5.60	0.30	0.390	8.01	4.59	0.05	0.360	6.32	4.98	0.11	0.360	3.28	4.47	0.24	0.390
Asp	0.45	2.55	0.43	0.440	3.21	1.99	0.06	0.370	2.21	2.18	0.16	0.370	1.90	2.06	0.18	0.370
tCr	-1.00	0.69	0.08	0.190	-0.88	0.65	0.09	0.190	-0.91	0.58	0.06	0.190	-1.91	0.67	0.00	0.030
GSH	3.61	1.17	0.00	0.010	2.83	1.37	0.02	0.050	1.95	1.83	0.15	0.170	2.62	1.47	0.04	0.070
Glu	-2.12	1.09	0.03	0.060	-2.06	0.83	0.01	0.030	-2.46	0.89	0.01	0.030	-2.72	1.01	0.01	0.030
Glx	-3.14	0.73	0.0001	0.0001	-3.59	0.79	<.0001	0.0001	-4.76	1.04	0.0001	0.0001	-4.77	0.88	<.0001	0.0001
Ins	-0.32	0.59	0.29	0.410	-0.03	0.58	0.48	0.480	-0.49	0.49	0.17	0.410	-0.92	0.73	0.11	0.410

Lac	-0.99	4.10	0.41	0.590	3.26	3.36	0.17	0.270	-1.00	3.78	0.40	0.490	-0.93	5.18	0.43	0.490
tNAA	-0.71	0.37	0.03	0.090	-0.67	0.41	0.06	0.110	-1.19	0.49	0.01	0.040	-1.18	0.48	0.01	0.040
tCh	-0.07	0.96	0.47	0.470	0.69	0.98	0.24	0.372	0.73	0.98	0.23	0.300	0.20	0.94	0.42	0.470
PE	-5.77	1.51	.0004	0.001	-6.73	2.10	0.002	0.002	-9.27	1.29	<.0001	<.0001	-9.34	1.62	<.0001	<.0001
Scyllo	-1.15	2.92	0.35	0.390	-1.96	2.92	0.25	0.390	-1.23	2.93	0.34	0.390	-4.94	2.93	0.05	0.210
Glc+Tau	-2.88	1.40	0.03	0.060	-2.84	1.46	0.03	0.060	-3.95	1.38	0.004	0.030	-2.58	1.49	0.05	0.070
sigma	1.90	1.90	0.16	0.290	1.88	2.04	0.18	0.290	1.21	2.19	0.29	0.330	1.42	2.55	0.29	0.330
Low dose																
	Block 1				Block 2				Block 3				Block 4			
	%	±SD	p	p_{FDR}	%	±SD	p	p_{FDR}	%	±SD	p	p_{FDR}	%	±SD	p	p_{FDR}
Asc	1.49	3.72	0.35	0.420	-1.14	3.64	0.38	0.420	0.93	3.34	0.39	0.420	-3.03	3.39	0.19	0.420
Asp	3.89	1.86	0.02	0.190	-1.48	1.86	0.22	0.290	0.34	2.26	0.44	0.440	2.24	2.16	0.16	0.250
tCr	0.18	0.38	0.32	0.430	1.20	0.41	0.004	0.030	1.06	0.50	0.02	0.090	0.48	0.46	0.15	0.240
GSH	1.63	1.74	0.18	0.250	1.46	1.82	0.22	0.250	1.89	1.94	0.17	0.250	1.17	1.71	0.25	0.250
Glu	0.67	0.65	0.16	0.320	2.21	1.02	0.02	0.150	1.60	0.86	0.04	0.150	0.14	0.86	0.43	0.490
Glx	-0.08	0.78	0.46	0.461	0.56	0.94	0.28	0.318	-0.51	0.80	0.27	0.318	-1.69	0.89	0.04	0.056
Ins	0.28	0.68	0.34	0.430	0.78	0.71	0.14	0.430	0.20	0.78	0.40	0.430	0.47	0.75	0.27	0.430
Lac	3.69	4.52	0.21	0.420	12.77	3.50	.0006	0.005	9.24	3.80	0.01	0.046	4.98	3.33	0.07	0.200
tNAA	0.19	0.21	0.18	0.210	0.63	0.31	0.03	0.110	0.46	0.19	0.01	0.100	0.36	0.24	0.07	0.150
tCh	0.11	0.77	0.44	0.440	0.46	0.65	0.24	0.320	1.44	0.69	0.02	0.070	0.37	0.81	0.33	0.370
PE	0.43	2.69	0.44	0.440	2.70	2.36	0.13	0.210	-2.10	2.25	0.18	0.210	2.09	2.21	0.18	0.210
Scyllo	0.43	2.83	0.44	0.440	-1.41	3.51	0.35	0.440	-3.77	3.10	0.12	0.440	-0.79	2.87	0.39	0.440
Glc+Tau	-1.67	1.34	0.11	0.290	-1.37	1.39	0.17	0.290	-1.83	1.42	0.10	0.290	-0.78	1.26	0.27	0.310
sigma	-3.12	1.00	0.002	0.019	-2.59	1.69	0.07	0.140	-0.35	1.87	0.43	0.500	-0.23	1.58	0.44	0.500
	Block 5				Block 6				Block 7				Block 8			
	%	±SD	p	p_{FDR}	%	±SD	p	p_{FDR}	%	±SD	p	p_{FDR}	%	±SD	p	p_{FDR}
Asc	2.29	2.87	0.22	0.420	-3.83	3.70	0.16	0.420	-0.79	3.74	0.42	0.420	3.23	4.30	0.23	0.420
Asp	2.07	1.91	0.14	0.250	3.51	2.46	0.08	0.250	0.76	1.90	0.35	0.400	3.28	2.58	0.11	0.250
tCr	0.92	0.52	0.05	0.120	0.60	0.49	0.12	0.230	0.19	0.67	0.39	0.430	-0.11	0.62	0.43	0.430
GSH	1.16	1.62	0.24	0.250	5.15	2.41	0.02	0.170	1.23	1.78	0.25	0.250	1.48	1.56	0.18	0.250
Glu	-0.02	0.82	0.49	0.490	-0.08	0.85	0.46	0.490	-0.57	1.09	0.30	0.480	-1.20	1.03	0.13	0.320

	%	±SD	p	pFDR	%	±SD	p	pFDR	%	±SD	p	pFDR	%	±SD	p	pFDR
Glx	-2.31	0.68	0.001	0.003	-3.30	0.82	0.0003	0.001	-2.88	0.88	0.002	0.003	-3.97	0.82	<.0001	0.003
Ins	0.88	0.69	0.11	0.430	0.16	0.88	0.43	0.430	0.39	0.82	0.32	0.430	-0.16	0.91	0.43	0.430
Lac	1.46	4.09	0.36	0.440	-2.20	4.05	0.30	0.440	0.85	3.50	0.41	0.440	-0.54	3.43	0.44	0.440
tNAA	0.35	0.24	0.08	0.150	0.11	0.28	0.34	0.340	-0.40	0.30	0.10	0.160	-0.35	0.37	0.17	0.210
tCh	1.86	0.85	0.02	0.070	1.69	0.82	0.03	0.070	1.24	1.03	0.12	0.240	1.04	1.01	0.16	0.250
PE	-3.93	2.73	0.08	0.210	-2.76	2.11	0.10	0.210	-3.14	2.86	0.14	0.210	-4.89	2.88	0.05	0.210
Scyllo	-1.89	2.88	0.26	0.440	0.57	3.41	0.43	0.440	-2.50	3.46	0.24	0.440	-5.37	3.58	0.07	0.440
Glc+Tau	-1.07	1.36	0.22	0.290	-1.01	1.04	0.17	0.290	-0.27	1.34	0.42	0.420	-1.26	1.58	0.22	0.290
sigma	-0.02	1.69	0.49	0.500	-1.79	1.79	0.16	0.260	-3.60	2.07	0.048	0.130	-3.94	2.16	0.04	0.130

High dose

	Block 1				Block 2				Block 3				Block 4			
	%	±SD	p	p_{FDR}	%	±SD	p	p_{FDR}	%	±SD	p	p_{FDR}	%	±SD	p	p_{FDR}
Asc	4.71	3.35	0.086	0.280	2.37	3.52	0.254	0.290	3.67	3.36	0.142	0.280	3.88	3.38	0.131	0.280
Asp	-3.67	1.51	0.011	0.088	-3.22	1.75	0.038	0.120	-2.52	1.58	0.062	0.120	-0.81	1.43	0.288	0.330
tCr	0.82	0.38	0.021	0.021	2.60	0.53	<.0001	.0002	2.05	0.65	0.002	0.004	2.16	0.52	0.0002	0.001
GSH	1.31	1.26	0.152	0.150	5.61	1.26	<.0001	<.0001	3.54	1.35	0.007	0.008	4.72	1.08	<.0001	0.0001
Glu	0.75	0.60	0.111	0.220	2.72	0.64	0.0001	0.001	2.00	0.66	0.003	0.010	2.26	0.80	0.004	0.012
Glx	0.82	0.63	0.10	0.116	2.27	0.76	0.003	0.006	0.94	0.69	0.003	0.116	-0.71	0.60	0.12	0.125
Ins	0.74	0.37	0.029	0.029	2.09	0.55	0.0004	0.001	1.67	0.54	0.002	0.004	2.86	0.70	0.0002	0.001
Lac	6.83	3.31	0.024	0.034	26.24	2.54	<.0001	<.0001	20.44	4.48	<.0001	0.0002	13.05	3.07	0.0001	0.0003
tNAA	0.02	0.20	0.470	0.047	0.79	0.28	0.004	0.012	0.39	0.30	0.101	0.160	0.72	0.29	0.010	0.019
tCh	1.73	0.66	0.007	0.007	5.13	0.75	<.0001	<.0001	3.50	1.12	0.002	0.002	3.52	0.95	0.0005	0.001
PE	-2.15	1.86	0.129	0.170	-5.16	1.33	0.0003	0.002	-1.66	2.43	0.250	0.250	-1.84	2.25	0.210	0.240
Scyllo	-0.42	2.42	0.432	0.490	1.77	2.55	0.246	0.480	1.97	2.44	0.213	0.480	2.47	2.48	0.164	0.480
Glc+Tau	-2.95	1.01	0.003	0.014	-3.35	1.01	0.001	0.010	-1.86	1.28	0.079	0.130	-2.81	1.27	0.018	0.048
sigma	-5.49	1.13	<.0001	0.0002	-6.45	1.91	0.001	0.003	-1.16	1.83	0.267	0.267	3.11	1.85	0.052	0.060
	Block 5				Block 6				Block 7				Block 8			
	%	±SD	p	p_{FDR}	%	±SD	p	p_{FDR}	%	±SD	p	p_{FDR}	%	±SD	p	p_{FDR}
Asc	5.21	3.40	0.068	0.280	3.91	4.81	0.211	0.290	1.55	3.92	0.348	0.350	3.48	5.04	0.248	0.290
Asp	0.06	2.16	0.489	0.490	1.41	1.77	0.216	0.290	3.08	1.90	0.058	0.120	1.70	1.58	0.146	0.120
tCr	1.88	0.58	0.001	0.004	1.62	0.53	0.003	0.004	1.35	0.53	0.009	0.010	1.33	0.52	0.009	0.010

GSH	5.68	1.17	<.0001	0.0001	5.46	1.09	<.0001	0.0001	5.29	1.17	<.0001	0.0001	4.25	1.35	0.002	0.003
Glu	0.66	0.83	0.214	0.280	0.48	0.67	0.241	0.280	-0.07	0.82	0.466	0.460	-0.74	0.85	0.195	0.280
Glx	-1.98	0.67	0.003	0.006	-2.25	0.80	0.004	0.007	-2.56	0.66	0.0003	0.002	-2.28	0.60	0.0004	0.002
Ins	2.12	0.77	0.005	0.007	2.11	0.55	0.0004	0.001	1.67	0.54	0.002	0.028	1.24	0.60	0.025	0.100
Lac	11.92	3.79	0.002	0.004	8.53	4.19	0.026	0.034	2.75	3.08	0.190	0.019	4.46	3.26	0.091	0.100
tNAA	0.74	0.26	0.004	0.012	0.67	0.19	0.001	0.007	0.15	0.29	0.309	0.035	0.17	0.22	0.228	0.030
tCh	4.03	0.95	0.0001	0.0002	3.70	0.87	0.0001	0.0002	3.70	0.97	0.0004	0.001	3.73	0.88	0.0001	0.0002
PE	-4.75	2.15	0.018	0.042	-4.48	2.21	0.026	0.042	-5.60	2.67	0.023	0.042	-4.18	1.91	0.019	0.042
Scyllo	-0.99	2.74	0.360	0.480	0.98	2.73	0.361	0.480	-1.41	2.52	0.290	0.480	-0.10	2.63	0.485	0.490
Glc+Tau	-2.69	1.47	0.039	0.078	-1.56	1.61	0.170	0.227	0.04	1.26	0.489	0.489	0.47	1.37	0.368	0.420
sigma	6.71	2.03	0.001	0.003	7.04	1.94	0.001	0.002	6.85	2.30	0.003	0.004	6.59	2.15	0.002	0.004

Asc: ascorbate; Asp: aspartate; Glc+Tau: glucose + composite taurine; Glu: glutamate; Glx: glutamate + glutamine; GSH: glutathione; Ins: inositol; Lac: lactate; PE: phosphoethanolamine; tCh: total choline; tCr: total creatine; tNAA: total N-acetylaspartate.

Table S18. phMRS: Dose-dependent effects of S-ketamine on neurometabolite levels in the ACC. Per comparison between conditions, and per infusion block, the % change with SD are provided, as well as the original p-value and the false discovery rate (FDR)-corrected p-values (p_{FDR}).

ACC: anterior cingulate cortex; phMRS: pharmacological magnetic resonance spectroscopy.

Placebo vs low dose

	Block 1				Block 2				Block 3				Block 4			
	%	±SD	p	p_{FDR}	%	±SD	p	p_{FDR}	%	±SD	p	p_{FDR}	%	±SD	p	p_{FDR}
Asc	7.62	13.15	0.28	0.405	-11.01	10.47	0.15	0.405	5.36	12.57	0.34	0.405	-9.01	11.32	0.22	0.405
Asp	80.55	62.70	0.11	0.494	-61.03	55.09	0.14	0.494	1.00	66.65	0.49	0.494	7.26	61.52	0.45	0.494
tCr	8.10	6.95	0.13	0.128	16.24	8.07	**0.03**	0.449	24.67	10.02	**0.01**	0.029	18.83	10.68	**0.05**	0.061
GSH	14.84	3.33	0.50	0.497	2841.7	1794.3	0.06	0.352	1544.4	1888.6	0.21	0.352	2106.1	1930.6	0.14	0.352
Glu	22.02	13.80	0.06	0.099	18.40	17.57	0.15	0.153	39.66	15.21	**0.01**	0.063	21.87	15.98	0.09	0.123
Ins	-0.96	10.16	0.46	0.463	8.47	10.80	0.22	0.294	12.87	12.83	0.16	0.261	13.04	12.29	0.15	0.261
Glx	95.8	45.94	0.46	0.484	28.18	37.07	**0.03**	0.112	42.28	38.72	0.08	0.146	35.95	39.37	0.08	0.141
Lac	-4.39	37.57	0.45	0.557	50.22	34.35	0.08	0.557	8.21	36.39	0.41	0.557	20.34	38.47	0.30	0.557
tNAA	7.52	5.65	0.10	0.098	16.24	7.32	**0.02**	0.074	11.71	7.67	0.07	0.095	17.88	10.21	**0.05**	0.093
tCh	-1.16	8.35	0.45	0.454	-2.36	5.09	0.32	0.432	7.62	7.65	0.16	0.432	1.44	8.99	0.44	0.445
PE	-7.84	19.93	0.35	0.399	-36.48	13.29	**0.01**	0.023	-16.67	15.25	0.14	0.190	-49.94	16.81	**0.01**	0.023
Scyllo	46.80	44.83	0.15	0.439	15.83	45.04	0.36	0.439	-11.28	45.23	0.40	0.439	70.45	45.35	0.07	0.439
Glc+Tau	-21.18	22.93	0.18	0.395	17.07	30.70	0.29	0.395	-8.85	27.05	0.37	0.395	12.50	23.56	0.30	0.395
sigma	-21.64	7.93	**0.01**	0.048	-30.76	12.93	**0.01**	0.052	-15.87	15.33	0.16	0.178	-18.77	12.95	0.08	0.107

	Block 5				Block 6				Block 7				Block 8			
	%	±SD	p	p_{FDR}	%	±SD	p	p_{FDR}	%	±SD	p	p_{FDR}	%	±SD	p	p_{FDR}
Asc	4.62	12.21	0.35	0.405	-24.59	13.65	0.339	0.34	-14.17	11.99	0.12	0.405	1.63	12.89	0.45	0.450
Asp	17.57	68.56	0.40	0.494	-1.76	65.93	0.494	0.49	-49.97	66.59	0.23	0.494	33.26	66.46	0.31	0.494
tCr	32.12	9.72	**0.002**	0.012	22.49	8.42	0.027	0.03	18.54	11.18	0.06	0.063	25.13	11.20	**0.02**	0.035
GSH	1817.3	1633.6	0.14	0.352	-1567.7	1995.1	0.352	0.35	803.44	1991.5	0.35	0.395	990.95	1728.6	0.29	0.381
Glu	39.47	20.45	**0.03**	0.079	31.48	13.94	0.068	0.07	32.30	17.54	**0.04**	0.079	22.92	19.95	0.13	0.150
Glx	47.04	37.29	0.06	0.146	65.51	42.58	0.21	0.141	11.46	40.89	**0.02**	0.082	66.20	36.02	0.18	0.226
Ins	20.42	14.82	0.09	0.261	4.68	13.91	0.423	0.42	17.76	11.57	0.07	0.261	16.20	15.11	0.15	0.261
Lac	21.03	39.51	0.30	0.557	-26.61	35.03	0.772	0.77	14.58	37.16	0.35	0.557	1.55	49.92	0.49	0.557
tNAA	23.15	9.25	**0.01**	0.074	18.44	9.48	0.085	0.09	15.54	10.86	0.08	0.095	17.34	11.46	0.07	0.095

tCh	19.50	7.73	**0.01**	0.076	10.95	7.64	0.331	0.33	5.18	9.86	0.30	0.432	6.60	9.28	0.24	0.432
PE	0.14	18.44	0.50	0.497	-20.98	16.06	0.164	0.16	-35.60	19.05	**0.04**	0.075	-31.31	15.11	**0.02**	0.066
Scyllo	-10.05	45.43	0.41	0.439	30.18	50.73	0.439	0.44	7.12	45.78	0.44	0.439	10.76	51.97	0.42	0.439
Glc+Tau	7.78	28.81	0.39	0.395	16.74	25.19	0.395	0.39	43.03	26.41	0.06	0.395	10.31	29.58	0.37	0.395
sigma	-7.81	11.80	0.26	0.257	-20.90	12.78	0.06	0.093	-33.79	17.01	**0.03**	0.061	-37.32	18.90	**0.03**	0.061

Placebo vs high dose

	Block 1				Block 2				Block 3				Block 4			
	%	±SD	p	p_{FDR}	%	±SD	p	p_{FDR}	%	±SD	p	p_{FDR}	%	±SD	p	p_{FDR}
Asc	22.69	23.81	0.18	0.38	-18.15	27.28	0.256	0.382	15.79	24.15	0.26	0.38	12.43	30.98	0.346	0.395
Asp	-12.39	10.04	0.12	0.44	-18.06	11.62	0.067	0.438	-10.97	11.44	0.17	0.44	-6.70	10.00	0.255	0.438
tCr	14.01	6.50	**0.02**	0.02	28.18	8.73	**0.002**	0.004	26.92	11.42	**0.01**	0.02	34.63	11.72	**0.004**	0.005
GSH	6.14	15.68	0.35	0.41	17.96	15.19	0.124	0.249	1.55	21.17	0.47	0.47	5.58	15.39	0.360	0.442
Glu	17.54	12.61	0.09	0.09	24.03	13.51	**0.044**	0.051	38.46	14.31	**0.01**	0.01	43.36	14.95	**0.004**	0.013
Glx	87.55	11.70	0.15	0.199	73.13	10.21	**0.004**	0.060	69.07	13.40	**0.02**	0.073	86.65	10.21	**0.008**	0.060
Ins	7.67	10.64	0.24	0.24	26.05	12.84	**0.027**	0.039	30.10	15.73	**0.03**	0.04	46.59	15.72	**0.003**	0.028
Lac	11.87	15.45	0.23	0.23	99.22	16.59	**<.0001**	<.0001	49.48	24.53	**0.03**	0.08	43.50	22.27	**0.032**	0.083
tNAA	4.40	8.73	0.31	0.31	27.62	9.60	**0.004**	0.007	12.99	12.46	0.15	0.18	37.08	13.59	**0.006**	0.008
tCh	12.98	10.87	0.12	0.12	50.92	12.93	**0.0003**	0.003	29.06	15.05	**0.03**	0.04	35.37	15.53	**0.016**	0.022
PE	10.32	37.33	0.39	0.45	31.20	27.34	0.133	0.338	-31.35	27.41	0.13	0.34	-34.26	36.52	0.179	0.338
Scyllo	14.61	27.20	0.30	0.33	32.11	27.42	0.127	0.243	43.15	29.68	0.08	0.21	93.55	27.60	**0.001**	0.010
Glc+Tau	-73.20	47.51	0.07	0.27	-26.42	57.34	0.325	0.389	-27.15	65.08	0.34	0.39	-47.67	58.71	0.213	0.340
sigma	-109.4	24.04	**<.0001**	0.0006	-181.1	43.92	**0.0002**	0.0008	-79.48	54.73	0.08	0.13	-5.40	51.45	0.459	0.459

	Block 5				Block 6				Block 7				Block 8			
	%	±SD	p	p_{FDR}	%	±SD	p	p_{FDR}	%	±SD	p	p_{FDR}	%	±SD	p	p_{FDR}
Asc	17.95	29.54	0.28	0.382	-32.44	24.19	0.096	0.382	-17.77	31.05	0.286	0.382	-1.98	31.25	0.475	0.475
Asp	-4.31	14.37	0.34	0.438	-0.36	10.02	0.486	0.486	4.87	12.84	0.354	0.438	4.11	10.04	0.343	0.438
tCr	36.38	11.01	**0.002**	0.004	32.80	10.52	**0.002**	0.004	30.45	9.52	**0.002**	0.004	41.23	10.43	**0.000**	0.003
GSH	18.75	15.43	0.118	0.249	35.70	18.04	**0.030**	0.177	32.69	18.39	**0.044**	0.177	9.62	15.49	0.270	0.412
Glu	40.54	16.07	**0.010**	0.015	37.66	11.85	**0.002**	0.013	38.25	13.64	**0.005**	0.013	34.78	14.31	**0.012**	0.016
Glx	85.65	10.80	0.10	0.158	80.50	11.64	**0.05**	0.141	68.71	13.27	**0.014**	0.073	69.63	10.83	**0.005**	0.060
Ins	37.36	18.78	**0.029**	0.039	31.82	14.63	**0.020**	0.039	31.44	12.38	**0.009**	0.037	31.15	16.17	**0.033**	0.039

Lac	43.06	23.75	**0.041**	0.083	21.94	22.00	0.164	0.230	18.94	19.62	0.172	0.230	18.38	24.75	0.233	0.233
tNAA	38.60	13.13	**0.004**	**0.007**	35.83	12.44	**0.004**	**0.007**	42.74	15.04	**0.005**	**0.007**	43.69	12.65	**0.001**	**0.007**
tCh	50.22	16.62	**0.003**	**0.012**	37.88	14.00	**0.006**	**0.016**	35.03	13.52	**0.008**	**0.016**	38.03	15.40	**0.011**	**0.017**
PE	18.04	36.10	0.311	0.415	-1.47	39.15	0.485	0.485	-27.04	33.08	0.211	0.338	-67.45	32.59	**0.025**	0.200
Scyllo	19.10	27.66	0.248	0.330	30.99	29.48	0.152	0.243	12.34	27.72	0.330	0.330	60.74	27.74	**0.019**	0.078
Glc+Tau	-60.39	60.21	0.163	0.326	9.24	72.33	0.450	0.447	106.59	69.21	0.069	0.274	60.15	57.86	0.155	0.326
sigma	73.07	54.44	0.096	0.128	75.92	53.87	0.086	0.128	87.36	65.00	0.096	0.144	77.33	65.68	0.126	0.144

Low dose vs high dose

	Block 1				Block 2				Block 3				Block 4			
	%	±SD	p	p_{FDR}	%	±SD	p	p_{FDR}	%	±SD	p	p_{FDR}	%	±SD	p	p_{FDR}
Asc	-5.17	18.74	0.393	0.480	-4.80	17.84	0.395	0.480	-3.46	20.37	0.433	0.480	-21.49	19.46	0.140	0.480
Asp	-42.20	14.86	**0.005**	**0.037**	-12.62	15.41	0.211	0.281	-20.72	16.07	0.105	0.281	-17.00	15.00	0.134	0.281
tCr	29.16	21.94	0.098	0.118	53.02	26.81	**0.030**	0.080	27.49	37.02	0.233	0.233	72.50	31.50	**0.015**	0.076
GSH	-1.96	15.61	0.451	0.451	45.85	19.18	**0.013**	**0.051**	17.47	18.98	0.183	0.245	29.74	18.39	0.060	0.106
Glu	12.97	49.36	0.398	0.398	33.60	72.48	0.324	0.398	23.48	67.92	0.366	0.398	118.83	65.33	**0.041**	0.328
Glx	78.12	18.25	0.121	0.182	67.67	21.21	0.071	0.146	69.75	21.37	0.085	0.146	81.62	16.47	0.138	0.195
Ins	67.56	98.01	0.249	0.249	126.83	135.0	0.179	0.204	148.49	126.6	0.126	0.204	295.94	137.8	**0.021**	0.170
Lac	-10.04	90.85	0.456	0.457	249.94	78.32	**0.002**	**0.016**	140.36	104.7	0.097	0.193	131.70	69.83	**0.036**	0.144
tNAA	30.00	38.91	0.224	0.299	-20.66	58.87	0.364	0.417	6.05	48.71	0.451	0.451	-59.02	52.77	0.137	0.272
tCh	-24.14	16.91	0.083	0.095	-80.52	17.66	**<.0001**	**0.001**	-29.27	27.53	0.149	0.149	-57.05	23.85	**0.013**	0.051
PE	-13.95	24.40	0.286	0.417	-66.61	20.43	**0.002**	**0.014**	-6.27	21.71	0.388	0.443	-43.63	26.31	0.055	0.222
Scyllo	3.82	110.8	0.486	0.486	126.40	123.7	0.159	0.311	238.74	120.2	**0.030**	0.140	176.38	112.4	0.065	0.174
Glc+Tau	30.33	33.82	0.190	0.309	49.50	34.59	0.083	0.221	25.66	34.44	0.232	0.309	65.29	34.03	**0.034**	0.221
sigma	28.18	13.84	**0.027**	**0.043**	54.59	22.50	**0.012**	**0.030**	27.25	21.55	0.109	0.125	-16.50	20.50	0.215	0.215
	Block 5				Block 6				Block 7				Block 8			
	%	±SD	p	p_{FDR}	%	±SD	p	p_{FDR}	%	±SD	p	p_{FDR}	%	±SD	p	p_{FDR}
Asc	-3.77	17.45	0.416	0.480	-23.76	23.76	0.164	0.480	-11.17	21.75	0.306	0.480	1.22	24.20	0.480	0.480
Asp	-17.30	19.77	0.195	0.281	-7.96	15.52	0.306	0.314	15.21	17.20	0.193	0.281	-9.35	19.03	0.314	0.314
tCr	40.68	31.25	0.103	0.118	47.39	27.89	0.051	0.103	47.74	33.46	0.084	0.177	70.39	32.00	**0.019**	0.076
GSH	38.61	18.85	**0.026**	0.070	13.94	21.22	0.259	0.296	42.59	16.73	**0.009**	0.051	25.66	16.46	0.066	0.106

Glu	33.54	58.97	0.288	0.398	40.36	56.24	0.240	0.398	31.03	66.52	0.323	0.398	63.41	67.45	0.178	0.398
Glx	99.55	16.51	0.489	0.489	89.23	18.21	0.280	0.320	94.64	16.52	0.374	0.408	66.51	19.38	**0.049**	0.141
Ins	161.45	123.9	0.103	0.204	225.22	130.4	**0.049**	0.195	117.46	124.5	0.178	0.204	150.13	142.2	0.151	0.204
Lac	107.00	71.37	0.074	0.193	118.47	104.4	0.134	0.215	11.67	82.66	0.444	0.457	28.88	79.15	0.359	0.457
tNAA	-44.08	45.20	0.170	0.272	-58.44	44.73	0.102	0.272	-97.41	61.07	0.062	0.249	-96.28	52.69	**0.040**	0.249
tCh	-36.83	24.13	0.070	0.095	-38.00	21.78	**0.047**	0.095	-39.61	27.58	0.082	0.095	-44.08	26.39	0.054	0.095
PE	-13.66	27.56	0.312	0.417	-24.20	21.41	0.135	0.360	-24.37	26.28	0.182	0.363	1.58	24.37	0.474	0.475
Scyllo	99.17	113.0	0.195	0.311	71.73	134.2	0.299	0.395	54.29	134.7	0.345	0.395	229.75	120.8	**0.035**	0.140
Glc+Tau	63.04	40.24	0.065	0.212	28.65	37.46	0.226	0.309	-6.89	44.94	0.440	0.440	-19.73	45.02	0.333	0.380
sigma	-43.20	23.37	**0.039**	0.052	-63.20	27.30	**0.015**	0.030	-87.41	34.39	**0.009**	0.030	-88.86	31.60	**0.005**	0.030

Asc: ascorbate; Asp: aspartate; Glc+Tau: glucose + composite taurine; Glu: glutamate; Glx: glutamate + glutamine; GSH: glutathione; Ins: inositol; Lac: lactate; PE: phosphoethanolamine; tCh: total choline; tCr: total creatine; tNAA: total N-acetylaspartate.

Table S19. Bayesian multilevel modeling to estimate condition based on individual-level phMRI contrast of parameter estimates in the ACC. A. Coefficients for the best-fitting Bayesian model with categorical outcome variable (condition) and phMRI COPE as predictor. Leave-one-out information criterion (LOO-IC): 1119.5±19.0; widely applicable information criterion (WAIC): 1119.5±19.0. B. Differences in predictor estimates extracted from the posterior distributions for low and high S-ketamine dose conditions. For all measures, the estimate and 95% confidence intervals are provided. Model: condition ~ COPE_fMRI + (1 | Subject)

ACC: anterior cingulate cortex; COPE: contrast of parameter estimate; phMRI: pharmacological magnetic resonance imaging.

A. Model estimates for low and high dose compared to placebo

	Placebo vs low dose	Placebo vs high dose
Multilevel hyperparameters		
intercept	Est: 0.09; CI: 0.00-0.25	Est: 0.10; CI: 0.00-0.30
Regression coefficients		
intercept	Est: -0.15; CI: -0.37-0.08	Est: -0.26; CI: -0.49 - -0.02
phMRI	Est: 0.03; CI: 0.02-0.04	Est: 0.04; CI: 0.03-0.05

B. Difference scores for high dose compared to low dose

	Low vs high dose
Regression coefficients	
phMRI	Est: 0.005; CI: -0.0007-0.011

Table S20. Bayesian multilevel modeling to estimate condition based on individual-level phMRS contrast of parameter estimates in the ACC. A. Coefficients for the best-fitting Bayesian model with categorical outcome variable (condition) and glutamate (Glu), lactate (Lac), aspartate (Asp), and glucose (Glc) COPE as predictors. LOO-IC: 1182.4±; WAIC: 182.3±15.5. **B.** Differences in predictor estimates extracted from the posterior distributions for low and high S-ketamine dose conditions. For all measures, the estimate and 95% confidence intervals are provided.

Model: condition ~ COPE_Glu + COPE_Asp + COPE_Lac + COPE_Glc + (1 | Subject)

ACC: anterior cingulate cortex; COPE: contrast of parameter estimate; phMRS: pharmacological magnetic resonance spectroscopy.

A. Model estimates for low and high dose compared to placebo		
	Placebo vs low dose	Placebo vs high dose
Multilevel hyperparameters		
intercept	**Est: 0.09; CI: 0.00-0.26**	**Est: 0.10; CI: 0.00-0.29**
Regression coefficients		
intercept	Est: 0.10; CI: -0.13-0.34	Est: -0.10; CI: -0.35-0.15
Glu	**Est: 41.63; CI: 24.26-59.59**	**Est: 41.35; CI: 23.73-59.77**
Lac	Est: -24.76; CI: -63.50-13.84	**Est: 42.60; CI: 2.99-81.66**
Asp	Est: 1.01; CI: -12.30-14.58	Est: -12.07; CI: -25.77-1.35
Glc	Est: 23.27; CI: -7.03-53.04	Est: 2.42; CI: -26.89-31.98

B. Difference scores for high dose compared to low dose	
	Low vs high dose
Regression coefficients	
Glu	Est: -0.27; CI: -18.39-17.74
Lac	**Est: 67.36; CI: 30.00-105.44**
Asp	Est: -13.08; CI: -26.70-0.38
Glc	Est: -20.86; CI: -49.88-7.12

Asp: aspartate; Glc: glucose + composite taurine; Glu: glutamate; Lac: lactate.

Table S21. Bayesian multilevel modeling to estimate condition based on individual-level combined phMRI-phMRS contrast of parameter estimates in the ACC. A. Coefficients for the best-fitting Bayesian model with categorical outcome variable (condition) and phMRI, glutamate (Glu), and lactate (Lac) COPE as predictors. LOO-IC: 1082.2±23.0; WAIC: 1082.1±23.0. **B.** Differences in predictor estimates extracted from the posterior distributions for low and high S-ketamine dose conditions. For all measures, the estimate and 95% confidence intervals are provided.

Model: condition ~ COPE_fMRI + COPE_Glu + COPE_Asp + COPE_Lac + COPE_Glc + (1 | Subject)

ACC: anterior cingulate cortex; COPE: contrast of parameter estimate; phMRI: pharmacological magnetic resonance imaging; phMRS: pharmacological magnetic resonance spectroscopy.

A. Model estimates for low and high dose compared to placebo		
	Placebo vs low dose	Placebo vs high dose
Multilevel hyperparameters		
intercept	**Est: 0.09; CI: 0.00-0.29**	**Est: 0.14; CI: 0.01-0.40**
Regression coefficients		
intercept	Est: -0.10; CI: -0.34-0.15	**Est: -0.41; CI: -0.67- -0.15**
phMRI	**Est: 0.03; CI: 0.02-0.04**	**Est: 0.04; CI: 0.03-0.05**
Glu	**Est: 39.40; CI: 21.35-58.05**	**Est: 43.60; CI: 24.04-62.92**
Lac	Est: -11.21; CI: -52.36-30.52	**Est: 56.96; CI: 15.26-100.02**

B. Difference scores for high dose compared to low dose	
	Low vs high dose
Regression coefficients	
phMRI	**Est: 0.006; CI: 0.0002-0.012**
Glu	Est: 4.20; CI: -13.00-21.37
Lac	**Est: 68.17; CI: 31.42-105.44**

Glu: glutamate; Lac: lactate.

Table S22. Association between subject-level neuroimaging measures and dissociation. A. Per infusion block, the results from the repeated measures correlations between the phMRI COPE values and total dissociation score, measured using the CADSS, are provided. **B.** Per infusion block, results from the repeated measures correlations between phMRS COPE values and total CADSS score per metabolite. COPE: contrast of parameter estimates; phMRI: pharmacological magnetic resonance imaging; phMRS: pharmacological magnetic resonance spectroscopy.

A. phMRI

	Block 1 (0 - 4 min)			Block 2 (4 – 8 min)			Block 3 (8 – 12 min)			Block 4 (12 – 16 min)		
	r	p	p_{FDR}	r	p	p_{FDR}	r	p	p_{FDR}	r	p	p_{FDR}
**phMRI**	.42	**.003**	**.005**	.70	**<.0001**	**<.0001**	.30	**.03**	**.03**	.36	**.01**	**.01**
	Block 5 (16 – 20 min)			Block 6 (20 – 24 min)			Block 7 (24 – 28 min)			Block 8 (28 – 32 min)		
	r	p	p_{FDR}	r	p	p_{FDR}	r	p	p_{FDR}	r	p	p_{FDR}
**phMRI**	.37	**.007**	**.01**	.48	**.0004**	**.001**	.49	**.0002**	**.001**	.37	**.008**	**.01**

B. phMRS

	Block 1 (0 - 4 min)			Block 2 (4 – 8 min)			Block 3 (8 – 12 min)			Block 4 (12 – 16 min)		
	r	p	p_{FDR}	r	p	p_{FDR}	r	p	p_{FDR}	r	p	p_{FDR}
**Glu**	0.33	**0.02**	0.08	0.30	**0.04**	0.08	0.31	**0.03**	0.08	0.25	0.08	0.11
**Asp**	-0.03	0.83	0.95	-0.22	0.13	0.86	-0.15	0.32	0.86	-0.13	0.39	0.86
**Glc**	-0.16	0.29	0.65	-0.02	0.88	0.88	0.02	0.88	0.88	0.09	0.55	0.88
**Lac**	-0.03	0.83	0.83	-0.20	0.17	0.83	-0.15	0.32	0.83	-0.13	0.39	0.83
	Block 5 (16 – 20 min)			Block 6 (20 – 24 min)			Block 7 (24 – 28 min)			Block 8 (28 – 32 min)		
	r	p	p_{FDR}	r	p	p_{FDR}	r	p	p_{FDR}	r	p	p_{FDR}
**Glu**	0.21	0.15	0.14	0.30	**0.04**	0.08	0.25	0.08	0.11	0.22	0.13	0.14
**Asp**	0.01	0.95	0.95	-0.10	0.48	0.86	-0.04	0.81	0.95	-0.09	0.36	0.86
**Glc**	0.03	0.83	0.88	0.14	0.33	0.65	0.33	**0.02**	0.16	0.18	0.21	0.65
**Lac**	-0.03	0.82	0.83	-0.10	0.49	0.83	-0.04	0.81	0.83	-0.09	0.54	0.83

Asp: aspartate; Glc: glucose + composite taurine; Glu: glutamate; Lac: lactate.

Table S22. Associations between phMRI outcomes and receptor distribution maps. The t-stat maps from the low and high S-ketamine dose compared to placebo were extracted and fed into a spin-test in neuromaps, to assess the correlation of these outcomes with relevant neurotransmitter receptor maps. To assess initial and delayed responses, both infusion blocks two and five were assessed for each condition and placebo-contrast.

**Low dose > placebo**				
	Block 2 (8 – 12 minutes)		Block 5 (16 – 20 minutes)	
	r	p	r	p
Glutamate MR5	0.40	**0.0099**	0.16	0.2277
NMDA	0.04	0.5446	0.04	0.5446
$GABA_A$	0.0	1.0	0.20	**0.0297**
NET	0.25	0.1782	0.04	0.8218
DAT	-0.03	0.7624	-0.10	0.2178
Dopamine D_2	-0.11	0.5347	-0.14	0.3681
VAChT	0.03	0.7822	-0.11	0.3960
mAChR M_1	0.07	0.5050	0.04	0.6535
5-HTT	-0.18	0.2673	-0.11	0.5545
5-HT2A	-0.05	0.5545	0.07	0.4455
Opioid μ	0.37	0.1980	-0.05	0.7822
Opioid κ	0.48	**0.0198**	0.07	0.7624
**High dose > placebo**				
	Block 2 (8 – 12 minutes)		Block 5 (16 – 20 minutes)	
	r	p	r	p
Glutamate MR5	0.40	**0.0099**	0.26	**0.0099**
NMDA	0.09	0.2871	0.07	0.3366
$GABA_A$	-0.03	0.7822	0.10	0.3069
NET	0.23	0.1980	0.07	0.7129
DAT	-0.02	0.9307	-0.05	0.6436
Dopamine D_2	-0.08	0.6733	-0.09	0.5911
VAChT	0.07	0.5545	-0.02	0.8317
mAChR M_1	0.02	0.8218	0.01	0.8614
5-HTT	-0.14	0.4752	-0.10	0.3069
5-HT2A	-0.02	0.7129	0.03	0.7030
Opioid μ	0.47	**0.0396**	0.19	0.4752
Opioid κ	0.52	**0.0099**	0.22	0.2673
**High dose > low dose**				
	Block 2 (8 – 12 minutes)		Block 5 (16 – 20 minutes)	
	r	p	r	p
Glu MR5	0.31	**0.0099**	0.29	**0.0099**
NMDA	0.05	0.4551	0.11	0.1782
$GABA_A$	-0.10	0.3069	0.04	0.7624
NET	0.15	0.4752	0.13	0.5446
DAT	-0.05	0.6436	-0.04	0.7327
Dopamine D_2	-0.07	0.6832	-0.02	0.9208

VAChT	0.03	0.8119	0.0	1.0
mAChR M_1	0.06	0.6139	0.09	0.3267
5-HTT	-0.11	0.5545	-0.06	0.7525
5-HT2A	0.02	0.7228	0.09	0.3069
Opioid μ	0.39	0.1683	0.20	0.4752
Opioid κ	0.35	0.0792	0.21	0.3069

Glu MR5: glutamate metabotropic receptor 5; NMDA: N-methyl-D-aspartate receptor; $GABA_A$: gamma-aminobutyric acid receptor A; NET: norepinephrine transporter; DAT: dopamine transporter; D_2: dopamine D_2 receptor; VAChT: acetylcholine transporter; mAChR M_1: metabotropic acetylcholine receptor 1; 5-HTT: serotonin transporter; 5-HT2A: serotonin 2A receptor.

Supplementary references

1. Esteban O, Markiewicz CJ, Blair RW, Moodie CA, Isik AI, Erramuzpe A *et al.* fMRIPrep: a robust preprocessing pipeline for functional MRI. *Nature Methods* 2019; **16**(1): 111-116.

   Esteban, Oscar, Ross Blair, Christopher J. Markiewicz, Shoshana L. Berleant, Craig Moodie, Feilong Ma, Ayse Ilkay Isik, et al. 2018. “FMRIPrep.” Software. Zenodo. https://doi.org/10.5281/zenodo.852659.

2. Gorgolewski K, Burns CD, Madison C, Clark D, Halchenko YO, Waskom ML, Ghosh SS. Nipype: a flexible, lightweight and extensible neuroimaging data processing framework in python. *Frontiers in Neuroinformatics* 2011; **5:** 13.

   Gorgolewski, Krzysztof J., Oscar Esteban, Christopher J. Markiewicz, Erik Ziegler, David Gage Ellis, Michael Philipp Notter, Dorota Jarecka, et al. 2018. “Nipype.” Software. Zenodo. https://doi.org/10.5281/zenodo.596855.

3. Tustison NJ, Avants BB, Cook PA, Zheng Y, Egan A, Yushkevich PA, Gee JC. N4ITK: improved N3 bias correction. *IEEE Trans Med Imaging* 2010; **29**(6): 1310-1320.

4. Avants BB, Epstein CL, Grossman M, Gee JC. Symmetric diffeomorphic image registration with cross-correlation: evaluating automated labeling of elderly and neurodegenerative brain. *Med Image Anal* 2008; **12**(1): 26-41.

5. Zhang Y, Brady M, Smith S. Segmentation of brain MR images through a hidden Markov random field model and the expectation-maximization algorithm. *IEEE Trans Med Imaging* 2001; **20**(1): 45-57.

6. Reuter M, Rosas HD, Fischl B. Highly accurate inverse consistent registration: a robust approach. *Neuroimage* 2010; **53**(4): 1181-1196.

7. Fonov V, Evans AC, Botteron K, Almli CR, McKinstry RC, Collins DL. Unbiased average age-appropriate atlases for pediatric studies. *Neuroimage* 2011; **54**(1): 313-327.

8. Evans AC, Janke AL, Collins DL, Baillet S. Brain templates and atlases. *Neuroimage* 2012; **62**(2): 911-922.

9. Jenkinson M, Smith S. A global optimisation method for robust affine registration of brain images. *Med Image Anal* 2001; **5**(2): 143-156.

10. Cox RW, Hyde JS. Software tools for analysis and visualization of fMRI data. *NMR Biomed* 1997; **10**(4-5): 171-178.

11. Greve DN, Fischl B. Accurate and robust brain image alignment using boundary-based registration. *Neuroimage* 2009; **48**(1): 63-72.

12. Pruim RHR, Mennes M, van Rooij D, Llera A, Buitelaar JK, Beckmann CF. ICA-AROMA: A robust ICA-based strategy for removing motion artifacts from fMRI data. *Neuroimage* 2015; **112:** 267-277.

13. Power JD, Mitra A, Laumann TO, Snyder AZ, Schlaggar BL, Petersen SE. Methods to detect, characterize, and remove motion artifact in resting state fMRI. *Neuroimage* 2014; **84:** 320-341.

14. Jenkinson M, Bannister P, Brady M, Smith S. Improved optimization for the robust and accurate linear registration and motion correction of brain images. *Neuroimage* 2002; **17**(2)**:** 825-841.

15. Behzadi Y, Restom K, Liau J, Liu TT. A component based noise correction method (CompCor) for BOLD and perfusion based fMRI. *Neuroimage* 2007; **37**(1)**:** 90-101.

16. Abraham A, Pedregosa F, Eickenberg M, Gervais P, Mueller A, Kossaifi J *et al*. Machine learning for neuroimaging with scikit-learn. *Front Neuroinform* 2014; **8:** 14.

17. Landheer K, Juchem C. Dephasing optimization through coherence order pathway selection (DOTCOPS) for improved crusher schemes in MR spectroscopy. *Magnetic Resonance in Medicine* 2019; **81**(4)**:** 2209-2222.

18. Landheer K, Swanberg KM, Juchem C. Magnetic resonance Spectrum simulator (MARSS), a novel software package for fast and computationally efficient basis set simulation. *NMR in Biomedicine* 2021; **34**(5)**:** e4129.

19. Tkáč I, Öz G, Adriany G, Uğurbil K, Gruetter R. In vivo 1H NMR spectroscopy of the human brain at high magnetic fields: Metabolite quantification at 4T vs. 7T. *Magn Reson Med* 2009; **62:** 868-879.

20. Virtanen P, Gommers R, Oliphant TE, Haberland M, Reddy T, Cournapeau D *et al*. SciPy 1.0: fundamental algorithms for scientific computing in Python. *Nat Methods* 2020; **17**(3)**:** 261-272.

21. Markello RD, Hansen JY, Liu Z-Q, Bazinet R, Shafiei G, Suárez LE *et al*. neuromaps: structural and functional interpretation of brain maps. *Nature Methods* 2022; **19**(11)**:** 1472-1479.

22. Cammoun L, Gigandet X, Meskaldji D, Thiran JP, Sporns O, Do KQ *et al*. Mapping the human connectome at multiple scales with diffusion spectrum MRI. *Journal of Neuroscience Methods* 2012; **203**(2)**:** 386-397.

23. Markello RD, Misic B. Comparing spatial null models for brain maps. *Neuroimage* 2021; **236:** 118052.

24. Alexander-Bloch AF, Shou H, Liu S, Satterthwaite TD, Glahn DC, Shinohara RT *et al*. On testing for spatial correspondence between maps of human brain structure and function. *NeuroImage* 2018; **178:** 540-551.

25. Burt JB, Helmer M, Shinn M, Anticevic A, Murray JD. Generative modeling of brain maps with spatial autocorrelation. *NeuroImage* 2020; **220:** 117038.
